# Effectiveness of Cellulose Sulfate Vaginal Gel for the Prevention of HIV Infection: Results of a Phase III Trial in Nigeria

**DOI:** 10.1371/journal.pone.0003784

**Published:** 2008-11-21

**Authors:** Vera Halpern, Folasade Ogunsola, Orikomaba Obunge, Chin-Hua Wang, Nneka Onyejepu, Oyinola Oduyebo, Doug Taylor, Linda McNeil, Neha Mehta, John Umo-Otong, Sakiru Otusanya, Tania Crucitti, Said Abdellati

**Affiliations:** 1 Family Health International, Research Triangle Park, North Carolina, United States of America; 2 College of Medicine, University of Lagos, Lagos, Nigeria; 3 Teaching Hospital, University of Port Harcourt, Port Harcourt, Nigeria; 4 HIV/STI Epidemiology and Control Unit, Institute of Tropical Medicine, Antwerp, Belgium; Tulane University, United States of America

## Abstract

**Background:**

This trial evaluated the safety and effectiveness of 6% cellulose sulfate vaginal gel in preventing male-to-female vaginal transmission of HIV, gonorrhea and chlamydial infection.

**Methods:**

This Phase III, double-blind, randomized, placebo-controlled trial was conducted between November 2004 and March 2007 in Lagos and Port Harcourt, Nigeria. We enrolled 1644 HIV-antibody negative women at high risk of HIV acquisition. Study participants were randomized 1∶1 to cellulose sulfate or placebo and asked to use gel plus a condom for each act of vaginal intercourse over one year of follow-up. The participants were evaluated monthly for HIV, gonorrhea and chlamydial infection, and for adverse events.

**Results:**

The trial was stopped prematurely after the data safety monitoring board of a parallel trial concluded that cellulose sulfate might be increasing the risk of HIV. In contrast, we observed fewer infections in the active arm (10) than on placebo (13), a difference that was nonetheless not statistically significant (HR = 0.8, 95% CI 0.3–1.8; p = 0.56). Rates of gonorrhea and chlamydial infection were lower in the CS group but the difference was likewise not statistically significant (HR = 0.8, 95% CI 0.5–1.1; p = 0.19 for the combined STI outcome). Rates of adverse events were similar across study arms. No serious adverse events related to cellulose sulfate use were reported.

**Conclusions:**

Cellulose sulfate gel appeared to be safe in the evaluated study population but we found insufficient evidence that it prevented male-to-female vaginal transmission of HIV, gonorrhea or chlamydial infection. The early closure of the trial compromised the ability to draw definitive conclusions about the effectiveness of cellulose sulfate against HIV.

**Trial Registration:**

ClinicalTrials.gov NCT00120770

## Introduction

As HIV has no cure, prevention technologies are needed to control the epidemic. The most effective current methods of HIV prophylaxis, including abstinence, male condoms and male circumcision, are partially or wholly controlled by men. The need for female-initiated methods for HIV prevention is all the more urgent, given the recent disappointing results from several HIV prevention trials among women [1]–[3] and the HIV vaccine field [4]. A safe, effective, and affordable topical microbicide could offer women a method to prevent sexual transmission of HIV in cases where other methods of protection cannot be used.

Cellulose sulfate (CS) has antimicrobial activity *in vitro* against various sexually transmitted pathogens, including HIV, *Neisseria gonorrhoeae* and *Chlamydia trachomatis* [5]. Prompted by the promising laboratory data and a good safety profile demonstrated in early-phase clinical trials [6]–[9], we proceeded to a Phase III study to evaluate the effectiveness of CS gel in preventing male-to-female vaginal transmission of HIV, gonorrhea and chlamydial infection among sexually active women perceived to be at high risk of HIV acquisition.

## Materials and Methods

The protocol for this trial and supporting CONSORT checklist are available as supporting information (see Checklist S1 and Protocol S1).

### Participants

This Phase III, double-blind, randomized, placebo-controlled trial was conducted in Lagos and Port Harcourt, Nigeria between November 2004 and March 2007. Three Institutional Review Boards approved the study: those of the College of Medicine, University of Lagos; the University of Port Harcourt Teaching Hospital; and Family Health International (FHI).

To be enrolled in the study women had to be HIV-seronegative, non-pregnant, 18–35-years-old and have, on average, three or more acts of intercourse per week and more than one sexual partner in the last three months. We excluded women who were injection drug users, were currently participating in another microbicide trial, were less than three months since their last pregnancy, or desired pregnancy in the next 12 months. Most study participants were low-income women who exchanged sex for money to supplement their incomes, although they did not self-identify exclusively as sex workers. All participants signed written informed consent form before screening and enrollment. Measures were put in place to ensure that the informed consent process was adequate for illiterate participants.

### Procedures

Each of the two study sites had two clinics with up to 15 outreach posts located in areas with densely concentrated, low-income populations where HIV transmission was thought to be high. The informal results of previous research among high risk populations as well as condom distribution programs conducted by local investigators and non-government organizations helped to identify such areas in Lagos and Port Harcourt. Outreach workers recruited women from bars, markets, and other common gathering areas and referred potential study participants to the study clinics for screening. After receiving detailed information about the study and signing the screening informed consent, women were screened for eligibility. An interview, general physical examination, pelvic examination, and testing for HIV, other sexually transmitted and reproductive tract infections (STIs and RTIs) and pregnancy were performed. During the interview baseline demographic data and information on medical history, contraceptive practices and sexual behavior were also collected. Risk reduction counseling, condom demonstration and free lubricated latex condoms not coated with nonoxynol-9 were provided to all participants. Eligible women were asked to return within 30 days for enrollment.

On return, participants signed an enrollment consent form and were then tested for HIV, syphilis, gonorrhea, chlamydial infection and pregnancy. Women who were pregnant or HIV positive were not enrolled. Women diagnosed with gonorrhea, chlamydial infection, trichomoniasis, syphilis, candidiasis or bacterial vaginosis during screening or enrollment were treated per the Centers for Disease Control and Prevention (CDC) treatment guidelines [10] and admitted to the study. Upon confirmation of eligibility, participants were randomized to the CS or placebo group and supplied with study gels and condoms. Study staff instructed participants to insert the contents of one full applicator of their assigned study gel into the vagina immediately prior to each act of sexual intercourse throughout the 12 months of study participation and reapply the gel if intercourse did not take place within one hour after application. Participants were instructed to use condoms for all acts of sexual intercourse regardless of gel use, not to douch after sex, not to use any other vaginal products, and not to use the study gel for anal intercourse. Participants were provided with referral information for local family planning clinics if they expressed interest in using contraceptives other than condoms.

Follow-up visits were held at the outreach post most conveniently located for each participant. Procedures included an interview, testing for HIV, gonorrhea, chlamydial infection and pregnancy, re-supply and demonstration - if required - of gel and condom use, and risk reduction and adherence counseling. As part of the interview process participants were asked about their health, any adverse experiences and concomitant medication use since their last visit, and coital frequency, gel and condom adherence in the last 7 days. Participants were encouraged to return for re-supply of condoms and gels if they ran out between their scheduled visits. Women presenting with an adverse event were referred to the study clinic for evaluation and treatment. Due to the investigational nature of the gel women who became pregnant stopped using product until the pregnancy had ended. To avoid social stigma, women that seroconverted were not discontinued from the study, nor did we require them to stop gel use so they could continue contributing to the STI and safety outcomes. All HIV-infected participants were referred to appropriate local facilities for social support and clinical management, including antiretroviral drugs if indicated.

### Study products

Both CS and placebo gels were identical in packaging and labeling and were administered in a 3.5 ml dose via a plastic single-use applicator. Each 3.5 ml application of 6% CS gel contained 231 mg of the active ingredient, sodium cellulose sulfate. The CS gel had a pH of 7.5. The placebo gel contained hydroxyethylcellulose (HEC) as a gelling agent, had no cell toxicity or anti-HIV properties, and had a pH of 4.4. The HEC placebo was previously deemed safe and sufficiently inactive for use in clinical studies of investigational microbicides [11].

### Outcomes

The primary outcome was incident HIV-1 or HIV-2 infection, as determined by antibodies in oral mucosal transudate using OraQuick® Advance Rapid HIV-1/2 Antibody test (OraSure Technologies, Inc., Bethlehem, PA, USA) and confirmed by Western blot (Genetic Systems™ HIV-1 Western Blot, Bio-Rad, Hercules, CA, USA). Western blot testing for HIV was carried out by the study laboratories in Nigeria. For women who seroconverted during first three months of follow-up, qualitative RNA-based polymerase chain reaction (PCR) testing for HIV with the AmpliScreen HIV-1 test (Roche Diagnostics, Branchburg, NJ, USA) was performed on stored enrollment plasma to assess whether the infection was pre-existing. PCR testing for HIV was also conducted on final visit plasma samples to identify recent infections in the absence of antibodies. HIV PCR testing was conducted by a laboratory of the Institute of Tropical Medicine (ITM, Antwerp, Belgium).

The secondary outcome was incident STI (gonorrhea or chlamydial infection), measured by detecting DNA material in self-administered vaginal swabs using the strand displacement amplification (SDA) BDProbeTec™ ET CT/NG assay (Becton Dickinson, Erembodegem, Belgium). The quality of SDA testing performed by the study laboratories in Nigeria was assured by repeat testing of all positive and 10% of all negative results from enrollment and quarterly follow-up visits at the ITM; in the event of a discrepancy the ITM result was used for data analysis. Storage and shipment of all biological samples were conducted in accordance with the ITM instructions, manufacturer's recommendations and requirements of a shipping company (World Courier, Allentown, PA, USA).

### Quality Assurance

The trial and its reporting complied with the CONSORT Guidelines [12]. We conducted the study under an Investigational New Drug application (IND) to the U.S. Food and Drug Administration and in accordance with Good Clinical Practice as established by the International Conference on Harmonisation [13]. The trial was registered with the ClinicalTrials.gov registry under #NCT00120770. The National Agency for Food and Drug Administration and Control of Nigeria approved the study prior to implementation.

### Sample Size

We aimed to enroll a total of 2,160 participants (1,080 in each treatment group) to observe 66 total HIV infections. This study size was designed to provide 80% power to detect a 50% reduction in the risk of HIV infection among CS gel users, controlling the type I error for falsely concluding a reduction in risk at the 0.025 level (an independent data and safety monitoring committee was to evaluate any potentially harmful effect of CS using less stringent criteria). A 50% reduction in typical use risk was considered to be a meaningful effect for impacting the epidemic. Such an assumption required that CS be more than 50% efficacious during consistent and correct use, since women would not use gel for all acts during the trial (e.g. due to withdrawal of product during pregnancy, missed product supply visits, and other non-adherence).

The sample size calculation assumed that loss to follow-up would not exceed 20% and that the incidence rate in the control group would be 5 per 100 woman-years. This rate of HIV infection was estimated based on HIV prevalence data available prior to study initiation [14]–[16], as well as incidence-to-prevalence ratios from previous research conducted by FHI among similar study populations in West Africa [17]. To compensate for the lack of directly measured incidence, we planned to monitor the overall infection rate during the study to determine if the sample size had to be adjusted to achieve the target number of events (66).

### Randomization and Blinding

Participants were randomly assigned to either the CS or placebo arm using a 1∶1 allocation ratio. A statistician not otherwise involved in the study developed the allocation sequence using a stratified (by study site), randomly permuted block design with block sizes 12, 18, and 24. Six product label colors (3 for CS and 3 for placebo) were used to improve blinding (revealing one color would not un-blind the entire study). Sequentially numbered, sealed opaque envelopes were used to assign participants to one of the six color groups after they signed the enrollment consent form and were determined eligible for the study. There was no indication that any unblinding occurred during the study.

### Statistical Methods

We compared the distribution of time to HIV infection between groups using an exact log-rank test, stratified by site. We calculated Kaplan-Meier estimates of HIV infection probabilities by treatment group, pooled across sites. Time to HIV infection, in days, was computed as the difference between the estimated date of HIV infection (based on the midpoint between the dates of the first confirmed positive HIV test visit and the preceding HIV negative visit) and the enrollment date, plus one. Data from participants who were lost to follow-up were included in the primary analysis but were censored on the date of their last HIV test visit. In secondary analyses, we used a proportional hazards regression model to estimate the hazard ratio (HR) for HIV infection, controlling for pre-specified baseline prognostic variables. The effect of CS gel in preventing transmission of gonorrhea and chlamydial infection was primarily evaluated by a proportional hazards model that controlled for site and other pre-specified baseline prognostic variables including age, history of pregnancy and anal intercourse, previous use of spermicides, number of male partners and sexual acts not protected by condoms, and positive results for gonorrhea or chlamydial infection at enrollment. We calculated exact confidence intervals for the relative risk of adverse events within system organ classes under a Poisson assumption for the event rates in each treatment group.

All primary analyses were performed on an intent-to-treat (ITT) basis, with the following modifications: randomized participants later found to be positive for HIV at enrollment (*via* HIV PCR testing) were excluded from analysis; and - for gonorrhea and chlamydial outcomes - women who were positive for STI at enrollment started their time in analysis on the date of their first negative SDA test following treatment.

We also performed pre-planned, exploratory on-product analyses of HIV, gonorrhea and chlamydial infection that excluded data collected from participants after their first documented interruption of product use (e.g. due to a positive pregnancy test, a lack of gel supplies following a missed visit, or safety concerns raised by the study clinician). Non-use of available product (e.g. choosing not to use available gel) was considered part of typical use and was not documented as a product interruption.

Data were collected at the sites on two-ply data collection forms and subsequently entered into FHI's 21 CFR Part 11 compliant Clintrial 4.5 database management system (Phase Forward, Inc., Waltham, MA, USA) by local data entry staff through a secure Internet server. Data analyses were implemented using version 9.1 of SAS statistical software (SAS Institute, Cary, NC, USA) and StatXact (Cytel Software, Cambridge, MA, USA).

Two interim analyses were planned. The first analysis took place after 16 HIV infections had occurred and focused exclusively on the safety profile of CS (i.e. there was no test of effectiveness). The second interim analysis was planned to occur after 33 infections to evaluate both safety and preliminary effectiveness data. We planned to use the Lan-Demets spending function [18] with O'Brien-Fleming type boundaries [19] to control the type I error for concluding effectiveness at the one-sided 0.025 level. In contrast, a fixed, one-sided p-value less than 0.10 in the direction of harm was used as the criteria to stop for potential harm or futility. Since the study was stopped prematurely due to external factors, no type I error adjustment was required when reporting the final results using two-sided p-values.

## Results

Study screening began in November 2004, and the last follow-up visit took place in March 2007. An independent Data Monitoring Committee (DMC) conducted the first planned interim safety analysis in September 2006 and their recommendation was to continue the study. An unplanned interim safety analysis was conducted on January 29, 2007 after an apparent increased risk of HIV in the CS arm was found in a parallel trial conducted by CONRAD, a cooperating agency of the United States Agency for International Development [20]. The DMC found no increased risk in the FHI study but recommended to stop due to the CONRAD study results.

### Participant Flow and Baseline Characteristics

**Figure 1:**
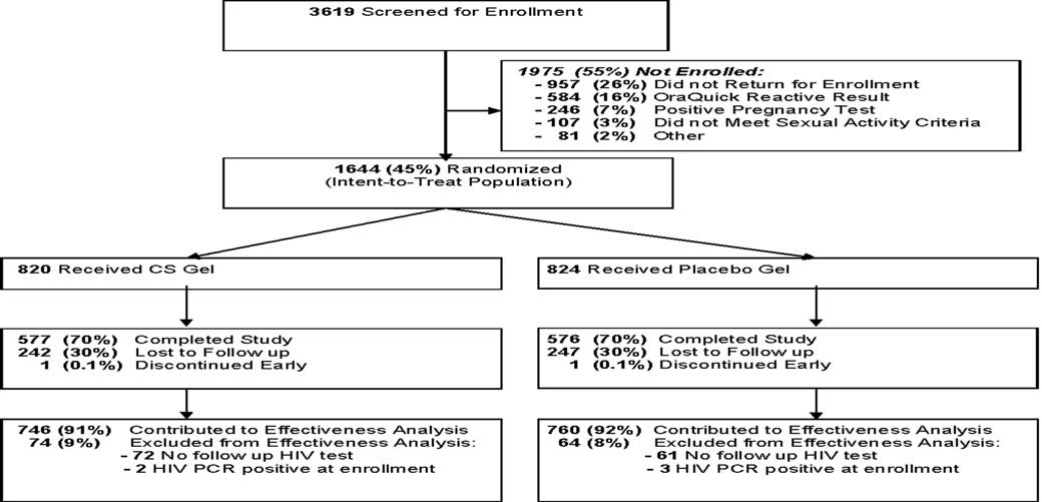
Participant Flow.

By the time of the early closure 3619 women had been screened, of whom 1644 (45%) were enrolled in the trial. Screening failures included women who did not return (26%), had a positive rapid HIV test (16%), were pregnant (7%) or met other exclusion criteria (6%). Participant's final status was similar in the two groups: 70% of women completed the study, 30% were lost to follow-up and an additional one woman in each group discontinued early (Figure 1). Eighteen percent of women missed one study visit, 9% missed two visits, and 38% missed 3 or more study visits. A mean of 293 days (median 357, range 16–380) were contributed to the effectiveness analysis, among those women with any follow-up. Demographic and baseline characteristics are presented in Table 1. The majority of the study participants were young, not married and relatively well educated. More than half of all women relied on condoms for pregnancy prevention prior to the trial, and more than half were diagnosed with bacterial vaginosis at baseline. The two groups appeared similar in all respects at baseline except for a slightly higher prevalence of chlamydial infection among those randomized to CS (4% vs. 2% on placebo; p = 0.045).

**Table 1:** Selected Baseline Characteristics by Treatment Group.

Characteristic^a^	CS (n = 820)	Placebo (n = 824)
Age in years (mean±SD^b^)	23.4±3.7	23.3±3.5
Not married (n, %)	780 (95%)	784 (95%)
Years of education (mean±SD)	10.2±3.8	10.4±3.6
Ever been pregnant (n, %)	654 (80%)	673 (82%)
Contraceptive method (n, %)		
none	170 (21%)	161 (20%)
hormonal	149 (18%)	143 (17%)
condom	451 (55%)	469 (57%)
other	50 (6%)	51 (6%)
Previous use of spermicide (n, %)	19 (2%)	26 (3%)
Douching (n, %)	578 (71%)	595 (72%)
Baseline STIs^c^ and RTIs^d^ (n, %)		
gonorrhea	57 (7%)	44 (5%)
chlamydial infection	32 (4%)	18 (2%)
syphilis	8 (1%)	6 (1%)
bacterial vaginosis	470 (57%)	461 (56%)
trichomoniasis	53 (6%)	45 (5%)
candidiasis	183 (22%)	204 (25%)
Number of different male sexual partners in the last 3 months (mean±SD)	20.8 (59.6)	17.8 (42.7)
Number of new male partners in the last 3 months (mean±SD)	17.4 (56.1)	14.5 (42.0)

a none of the differences between the study groups were statistically significant except for prevalence of chlamydial infection (p = 0.045, unadjusted for multiple comparisons).

b Standard Deviation.

c Sexually Transmitted Infections.

d Reproductive Tract Infections.

### Sexual Behavior and Product use

Self-reported sexual behaviors were similar between groups (Table 2). The average coital frequency increased slightly during the first month of the study but returned to enrollment levels at month 12, whereas the average reported number of sexual partners and new partners in the last 30 days decreased from enrollment to month 12. Self-reported condom use increased from screening (60%) to enrollment (89%), and fluctuated little during follow-up (84–90%). On average, gel was reportedly used in 81% of all sexual acts, and in approximately 50% of sexual acts not protected by condoms.

**Table 2:** Self-reported Sexual Behavior, Gel and Condom Use by Treatment Group*.

Treatment	Visit	In the previous 30 days		In the previous 7 days			
		Number of male partners Mean (SE^a^)	Number of new partners Mean (SE)	Number of vaginal sex acts Mean (SE)	% of sex acts using condom	% of sex acts using gel	% of condom-free sex acts using gel^b^
**CS (N = 820)**	Screening	Data not collected	Data not collected	6.0 (0.3)	61.2^c^	Not applicable	Not applicable
	Enrollment	10.1 (0.8)	5.8 (0.8)	6.2 (0.3)	88.7	Not applicable	Not applicable
	Month 1	10.8 (0.7)	4.7 (0.4)	8.5 (0.4)	89.0	84.2	52.0
	Month 6	9.4 (0.6)	4.1 (0.4)	7.8 (0.4)	89.6	80.6	45.4
	Month 12	7.0 (0.9)	2.9 (0.7)	5.1 (0.3)	84.3	76.0	51.4
**Placebo (N = 824)**	Screening	Data not collected	Data not collected	5.4 (0.2)	60.2^c^	Not applicable	Not applicable
	Enrollment	9.4 (0.9)	4.3 (0.5)	5.9 (0.2)	88.6	Not applicable	Not applicable
	Month 1	12.2 (1.2)	4.7 (0.5)	8.0 (0.4)	87.6	81.3	52.9
	Month 6	10.6 (1.0)	4.7 (0.5)	8.2 (0.4)	87.2	80.4	47.1
	Month 12	7.8 (0.9)	3.6 (0.7)	5.8 (0.3)	88.8	77.9	56.5

* none of the differences between the study groups were statistically significant.

a Standard Error.

b estimated based on self-reported adherence data.

c estimated as % of condom use in the last vaginal sex at screening.

A total of 645 product interruptions were documented during the trial (323 on CS and 322 on placebo). In both groups pregnancy was the primary reason for product discontinuation (54%), followed by a lack of supplies due to missed visits (45%). Despite the high pregnancy rates (29 and 28 per 100 woman-years in the CS and placebo groups, respectively; Table 3), only 4.8% of total observed person-time was off product due to pregnancy (5.1% for CS and 4.5% for placebo) because most women did not carry their pregnancy to term.

**Table 3:** Incidence of HIV, Gonorrhea and Chlamydial Infection by Treatment Group.

Outcome	CS				Placebo				Hazard Ratio (95% CI)
	No. of Women	Woman-Years	No. of Events	Event Rate*	No. of Women	Woman-Years	No. of Events	Event Rate*	
**HIV**	746	599	10	1.7	760	609	13	2.1	0.8 (0.3, 1.8)
**Gonorrhea**	673	533	37	6.9	679	529	52	9.8	0.7 (0.5, 1.1)
**Chlamydia**	673	547	15	2.7	686	553	22	4.0	0.7 (0.4, 1.3)
**Gonorrhea or Chlamydia**	670	522	50	9.6	675	521	64	12.3	0.8 (0.5, 1.1)
**Pregnancy**	746	531	156	29	760	541	152	28	1.05 (0.8, 1.3)

* per 100 woman-years.

Viewed by site, women in Port Harcourt were better educated, more likely to be students, less likely to be married, and reported more oral sex and less contraceptive use at baseline than in Lagos. In addition, participants in Port Harcourt reported higher risk of sexual behavior and used more gel than women in Lagos. They also were more adherent with gel use and more compliant with the protocol (data not shown).

### Effect on HIV, Gonorrhea and Chlamydial infection

A total of 1506 women contributed data to the primary HIV analysis, of whom 23 had an incident infection (pooled incidence rate of 1.9 per 100 woman-years). All incident infections were diagnosed as HIV-1. Fewer infections occurred in the CS group (10) than on placebo (13), but the difference was not statistically significant (HR = 0.8, 95% CI 0.3–1.8, p = 0.56; Table 3). Premature closure of the trial greatly curtailed the power of the study to detect an effect on HIV (23 events provided only ∼40% power to detect a 50% reduction in risk). In contrast, we observed a total of 126 incident gonorrhea and chlamydial infections which provided greater than 90% power to detect a 50% reduction in the risk of acquiring an STI among women randomized to CS. Although the rates of both STIs were lower in the CS group, the difference was not statistically significant (HR = 0.8, 95% CI 0.5–1.1; p = 0.19 for the combined STI outcome; Table 3).

We excluded data from participants after a documented product interruption in planned secondary analyses in order to estimate effectiveness among women who had product available for use (we did not exclude self-reported imperfect use because self-reported adherence is subject to unknown levels and directions of bias). This exclusion resulted in only a slight change to the HIV result (HR 0.9, 95% CI 0.4–2.3; p = 0.86), the combined STI result (HR = 0.7, 95% CI 0.5–1.1; p = 0.15) and the chlamydial infection result (HR 0.8, 95% CI 0.3–1.8; p = 0.57), whereas the rate of gonorrhea was lower in the CS group (HR 0.6, 95% CI 0.4–1.0; p = 0.058). Additional, unplanned exploratory analyses suggested that CS use may have been associated with lower risk of gonorrhea or chlamydial infection among women who reported above median coital frequency at baseline (HR 0.4, 95% CI 0.2–0.8; p = 0.01) and above median average number of partners during follow up (HR 0.6, 95% CI 0.4–1.0; p = 0.05).

We also explored the effects of other covariates on the risk of HIV infection, irrespective of treatment group. Previous experience using spermicides was associated with higher risk of HIV (HR 6.7, 95% CI 1.8–4.7; p<0.01), although less than 3% of women reported previous spermicide use. Similarly, a positive gonorrhea or chlamydial infection result at baseline was associated with higher risk of both HIV (HR 3.6, 95% CI 1.2–10.8; p = 0.02) and gonorrhea or chlamydial infection (HR 2.5, 95% CI 1.2–5.2; p = 0.01).

### Safety

A total of 47 serious adverse events (SAEs) were identified, but none were related to gel use. Hospitalization due to malaria and typhoid were the most frequent SAEs. Overall adverse event rates, including bacterial vaginosis and genital pruritus - the two most common reproductive system disorders - were generally the same among study groups (Table 4). Most of the adverse events were mild and resolved with no sequelae. Rates of reported intermenstrual bleeding were similar between groups.

**Table 4:** Adverse Experiences Reported in at least 5% of Women by Treatment Group.

System Organ Class Category*	Preferred Term	CS (N = 750)				Placebo (N = 763)				CS vs. Placebo
		Number of Events	Number of Women	Percent of Women	IR**	Number of Events	Number of Women	Percent of Women	IR**	Rate Ratio (95% CI)
**Infections and infestations**	Malaria	215	169	22.5	31.8	207	160	21.0	29.1	1.1 (0.9, 1.4)
**Gastrointestinal disorders**	Abdominal pain	96	73	9.7	12.6	127	102	13.4	17.6	0.7 (0.5, 1.0)
**Nervous system disorders**	Headache	67	58	7.7	9.7	80	68	8.9	11.5	0.8 (0.6, 1.2)
**General disorders and administration site conditions**	Pyrexia	59	52	6.9	8.7	49	46	6.0	7.5	1.2 (0.8, 1.8)
**Respiratory, thoracic and mediastinal disorders**	Respiratory tract infection	65	52	6.9	8.7	72	63	8.3	10.5	0.8 (0.6, 1.2)
**Reproductive system and breast disorders**	Vaginitis bacterial	88	74	9.9	12.4	98	77	10.1	12.9	1.0 (0.7, 1.3)
	Genital pruritus female	82	70	9.3	11.8	78	65	8.5	10.7	1.1 (0.8, 1.6)
	Vaginal candidiasis	52	43	5.7	7.1	65	51	6.7	8.4	0.8 (0.6, 1.3)
	Menstrual disorder	51	41	5.5	6.6	56	48	6.3	7.9	0.8 (0.5, 1.3)
	Vaginal discharge	35	31	4.1	4.9	39	38	5.0	6.2	0.8 (0.5, 1.3)

* MedDRA™ coding terminology was used for coding adverse events.

** Incidence rate per 100 woman-years.

## Discussion

Early termination of the trial compromised our ability to draw definitive conclusions about the effectiveness of CS. However, our final HIV result (HR = 0.8, 95% CI 0.3–1.8) was inconsistent with the size of the interim effect of a parallel trial conducted by CONRAD that led to premature closure of both CS studies (estimated HR = 2.2) [20] as well as with the direction of the final ITT result from that study (HR 1.6; 95% CI 0.9–3.0). [21] If CS is indeed associated with increased risk of HIV, it may be so only at high rates of gel use like those reported in two of the CONRAD trial sites (mean of more than 20 times per week in Uganda and Benin), [21] compared to only 8 per week on average in the Nigeria trial. The potential for extremely high frequencies of gel use in some populations may indicate the need for Phase I safety studies that go beyond the standard once- or twice-daily application for 14 days.

Vaginal douching was reported at baseline by 70% of the study participants in Nigeria. If CS does increase risk of acquiring HIV, one could argue that vaginal cleansing might have removed the gel and masked any ill effects in the Nigeria trial. However, all study participants were instructed not to douche after gel application and sex, and douching was only reported to have taken place at 6% of recorded follow-up visits.

Self-reported condom use was considerably higher during the trial than at screening. While this may be entirely due to social desirability bias, it may also suggest that our counseling messages, among other factors, modified the sexual behavior of participants, which could in turn explain the lower than expected incidence of HIV in both treatment groups. Although effective risk reduction counseling and condom promotion has positive public health implications, substantially larger study sizes may be required to achieve adequate numbers of HIV endpoints in a clinical trial.

Although less than expected when designing the study, the overall incidence of HIV of 1.9 per 100 woman-years was relatively high considering that the overall prevalence of HIV in Nigeria does not exceed 6% [14]. For comparison, Padian et al. reported an HIV incidence of 2.7 per 100 woman-years in Zimbabwe [1], a country where the HIV epidemic is more generalized and national adult HIV prevalence exceeds 15% [22]. Targeting women at highest risk of HIV took a heavy toll on study retention however: we compromised our follow up rates by recruiting young women at highest risk of HIV infection, residential mobility, and poor compliance with the protocol procedures. Despite the rigorous efforts of in-country study teams, one third of participants in each group were lost.

Since rates of loss to follow-up were non-differential between the two study groups, and since baseline characteristics of women who were lost to follow-up were similar between the CS and placebo group (data not shown), there is no evidence that our estimate of treatment effect was biased. Nonetheless, high loss to follow-up compromises the interpretation of study findings. The fact that our retention rates were in line with those reported in similar study populations emphasizes the challenges associated with performing clinical research among high risk women in resource poor settings [23], [24].

Although we observed very high pregnancy rates, relatively few of the pregnancies were carried to term and only 5% of woman-years of follow-up were off product due to pregnancy. Moreover, excluding data collected after product interruptions did not influence the primary effectiveness result. Thus we have no evidence that our estimate of treatment effect was confounded by high pregnancy rates. Nonetheless, preventing pregnancies in future trials remains an important task in mitigating safety concerns and ensuring validity of results [25].

In spite of the satisfactory self-reported overall rate of adherence to gel (more than 80% of sex acts covered in each group), we estimated that gel was used for only 50% of acts when a condom was not used. Had the study continued to the planned total of 66 endpoints, such a level of adherence would have seriously compromised the power to detect an effect of CS. Given the strong contraceptive profile of CS [26], the lack of an observed difference in pregnancy rates between groups is disappointing, and provides additional evidence of possible poor adherence to study product. Previously reported differential use of prevention methods with different partner types [27] might account for the overall low use of gel among acts without condoms. As we did not collect information on partner type, this question remains unanswered.

Strengths of our trial included accurate detection of incident infections, as well as close monitoring and proactive management of the recruitment facilitated by remote data entry. Unfortunately the early closure of the trial, poor retention, and possible low adherence impaired our ability to detect an effect – positive or negative - of CS. The trial highlighted many of the challenges associated with the evaluation of candidate microbicides, but we hope that the methodological lessons learned and knowledge gained in Nigeria will be useful to researchers planning future HIV prevention trials.

## Supporting Information

Checklist S1 CONSORT Checklist
(0.06 MB DOC)

Protocol S1 Trial Protocol
(1.21 MB DOC)
