# Supplementary material for: Effectiveness of Cellulose Sulfate Vaginal Gel for the Prevention of HIV Infection: Results of a Phase III Trial in Nigeria
Source: PLoS One. 2008 Nov 21;3(11):e3784. doi: 10.1371/journal.pone.0003784 (PMC2582655; doi:10.1371/journal.pone.0003784)
Supplement: Protocol S1 — Trial Protocol (1.21 MB DOC) [file pone.0003784.s002.doc]

# **Randomized Controlled Trial of Cellulose Sulfate Gel and HIV in Nigeria**

Study 9757

**Funded by:** US Agency for International Development

**Sponsor:** CONRAD

1611 North Kent Street, Suite 806

Arlington, VA 22209

**Investigational Product:** Sodium Cellulose Sulfate (CS) 6%

IND No. 69,107

**Monitor:** Family Health International

PO Box 13590

Research Triangle Park, NC 27709

**FHI Project Leader:** Vera Halpern, MD

Scientist I

PO Box 13590

Research Triangle Park, NC 27709

Tel: 1.919.544.7040

**FHI Chief Medical Officer:** David Grimes, MD

Vice-President of Biomedical Affairs

PO Box 13590

Research Triangle Park, NC 27709

Tel: 1.919.544.7040

**FHI Study Clinician:**  Elizabeth Raymond, MD

Scientist II

PO Box 13590

Research Triangle Park, NC 27709

Tel: 1.919.544.7040

**Investigators:** Folasade T. Ogunsola, MD

Senior Lecturer, Dept of Medical Microbiology and Parasitology
College of Medicine, University of Lagos
PMB 12003 Lagos

Tel: 234-1-7764717

Lagos, Nigeria

Orikomaba Obunge, MD

Head, Dept of Medical Microbiology and Parasitology

University of Port Harcourt Teaching Hospital

PMB 6173 Port Harcourt

Tel: 234-084-486709

Port Harcourt, Nigeria

**Clinical Labs:** Central Lab, College of Medicine, University of Lagos

Central Lab, University of Port Harcourt Teaching Hospital

# **Randomized Controlled Trial of Cellulose Sulfate Gel and HIV in Nigeria**

Study 9757

I, the Investigator, agree to conduct this study in full accordance with the provisions of this protocol and will comply with all requirements regarding the obligations of clinical Investigators as fully outlined in the Declaration of Helsinki and in the Statement of Investigator, which I have also signed. I agree to maintain all study documentation until Family Health International (FHI) advises that it is no longer necessary. I also agree to publish or present data only upon review and after discussion with FHI.

I have read and understand the information in this protocol, including the potential risks and side effects of the product under investigation, and will ensure that all associates, colleagues, and employees assisting in the conduct of the study are informed about the obligations incurred by their contribution to the study

__________________________________ __________________________________

Signature of Investigator Date

Prepared by:

Clinical Research Division

Family Health International

P.O. Box 13950

Research Triangle Park, NC 27709 USA

2224 E. NC Highway 54

Durham, NC 27713 USA

Tel: 1-919-544-7040

FAX: 1-919-544-7261

TABLE OF CONTENTS

[STUDY SUMMARY 6](#__RefHeading___Toc107045782)

[LIST OF ABBREVIATIONS AND ACRONYMS 7](#__RefHeading___Toc107045783)

[1.0 INTRODUCTION 8](#__RefHeading___Toc107045784)

[1.1 Background 8](#__RefHeading___Toc107045785)

[1.2 Preclinical Studies 8](#__RefHeading___Toc107045786)

[1.3 Completed Clinical Studies 9](#__RefHeading___Toc107045787)

[1.4 Ongoing and Planned Clinical Studies 10](#__RefHeading___Toc107045788)

[1.5 Rationale 10](#__RefHeading___Toc107045789)

[2.0 STUDY OBJECTIVES 11](#__RefHeading___Toc107045790)

[2.1 Primary Objective 11](#__RefHeading___Toc107045791)

[2.2 Secondary Objectives 11](#__RefHeading___Toc107045792)

[3.0 TRIAL DESIGN 11](#__RefHeading___Toc107045793)

[3.1 Primary Endpoint 11](#__RefHeading___Toc107045794)

[3.2 Secondary Endpoints 11](#__RefHeading___Toc107045795)

[3.3 Study Design 11](#__RefHeading___Toc107045796)

[3.4 Measures to Minimize Bias 12](#__RefHeading___Toc107045797)

[3.4.1 Randomization 12](#__RefHeading___Toc107045798)

[3.4.2 Allocation concealment 13](#__RefHeading___Toc107045799)

[3.4.3 Blinding 13](#__RefHeading___Toc107045800)

[3.4.4. Randomization codes and decoding procedure 13](#__RefHeading___Toc107045801)

[3.5 Duration of the Study 13](#__RefHeading___Toc107045802)

[3.6 Discontinuation Criteria 14](#__RefHeading___Toc107045803)

[3.6.1 Individual participants 14](#__RefHeading___Toc107045804)

[3.6.2 Study discontinuation 15](#__RefHeading___Toc107045805)

[3.7 Study Product Accountability 15](#__RefHeading___Toc107045806)

[3.8 Data Directly Recorded on DCFs 15](#__RefHeading___Toc107045807)

[4.0 SELECTION AND WITHDRAWAL OF PARTICIPANTS 15](#__RefHeading___Toc107045808)

[4.1 Eligibility Criteria 15](#__RefHeading___Toc107045809)

[4.2 Completion 16](#__RefHeading___Toc107045810)

[4.3 Loss to Follow-up 16](#__RefHeading___Toc107045811)

[5.0 STUDY PRODUCT AND TREATMENTS 17](#__RefHeading___Toc107045812)

[5.1 CS Gel 17](#__RefHeading___Toc107045813)

[5.2 Placebo Gel 17](#__RefHeading___Toc107045814)

[5.3 Gel Labeling 17](#__RefHeading___Toc107045815)

[5.4 Concomitant Treatments 18](#__RefHeading___Toc107045816)

[5.5 Monitoring of Product Compliance 18](#__RefHeading___Toc107045817)

[6.0 EFFECTIVENESS ASSESSMENT 18](#__RefHeading___Toc107045818)

[6.1 Effectiveness Parameters 18](#__RefHeading___Toc107045819)

[6.1.1 HIV effectiveness 18](#__RefHeading___Toc107045820)

[6.1.2 GC or CT effectiveness 18](#__RefHeading___Toc107045821)

[6.2 Methods and Timing of Effectiveness Parameters 19](#__RefHeading___Toc107045822)

[6.2.1 HIV effectiveness 19](#__RefHeading___Toc107045823)

[6.2.2 GC and CT effectiveness 20](#__RefHeading___Toc107045824)

[7.0 ADVERSE EVENTS AND REPORTING REQUIREMENTS 20](#__RefHeading___Toc107045825)

[7.1 Adverse Events 20](#__RefHeading___Toc107045826)

[7.2 Relationship of AE to study product 21](#__RefHeading___Toc107045827)

[7.3 Reporting AEs 22](#__RefHeading___Toc107045828)

[7.4 Serious Adverse Events 22](#__RefHeading___Toc107045829)

[7.5 Reporting SAEs 23](#__RefHeading___Toc107045830)

[7.6 Social Risk Events 23](#__RefHeading___Toc107045831)

[8.0 STUDY VISIT SUMMARY 24](#__RefHeading___Toc107045832)

[8.1 Screening Visit 24](#__RefHeading___Toc107045833)

[8.2 Enrollment Visit 25](#__RefHeading___Toc107045834)

[8.3 Follow-up Visits 27](#__RefHeading___Toc107045835)

[8.4 Interim Contacts and Unscheduled Visits 28](#__RefHeading___Toc107045836)

[9.0 STATISTICAL SUMMARY 28](#__RefHeading___Toc107045837)

[9.1 Study Size Justification 28](#__RefHeading___Toc107045838)

[9.2 Analysis Plan Summary 28](#__RefHeading___Toc107045839)

[9.2.1 Analysis of effectiveness outcomes 29](#__RefHeading___Toc107045840)

[9.2.2 Analysis of safety outcomes 30](#__RefHeading___Toc107045841)

[9.3 Interim Analysis Plan Summary 30](#__RefHeading___Toc107045842)

[9.3.1 Study size assessment 31](#__RefHeading___Toc107045843)

[9.3.2 Interim analysis of HIV outcome 31](#__RefHeading___Toc107045844)

[10.0 MONITORING PLAN SUMMARIES 31](#__RefHeading___Toc107045845)

[10.1 Clinical Monitoring Plan 31](#__RefHeading___Toc107045846)

[10.2 Data Monitoring Committee 32](#__RefHeading___Toc107045847)

[11.0 DATA MANAGEMENT PLAN SUMMARY 32](#__RefHeading___Toc107045848)

[12.0 PROTOCOL VIOLATIONS 33](#__RefHeading___Toc107045849)

[13.0 STUDY DOCUMENTS 33](#__RefHeading___Toc107045850)

[13.1 Study Initiation 33](#__RefHeading___Toc107045851)

[13.2 Study Conduct 35](#__RefHeading___Toc107045852)

[13.3 Study Completion 36](#__RefHeading___Toc107045853)

[13.4 Site Record Retention and Access to Documents at the Site 36](#__RefHeading___Toc107045854)

[14.0 QUALITY ASSURANCE 36](#__RefHeading___Toc107045855)

[15.0 ETHICS AND RESEARCH INTEGRITY 37](#__RefHeading___Toc107045856)

[15.1 Institutional Review Board Review and Approval 37](#__RefHeading___Toc107045857)

[15.2 Informed Consent 37](#__RefHeading___Toc107045858)

[15.3 Participant Confidentiality 37](#__RefHeading___Toc107045859)

[15.4 Research Integrity 37](#__RefHeading___Toc107045860)

[16.0 PUBLICATION POLICY 38](#__RefHeading___Toc107045861)

[17.0 ADDITIONAL INVESTIGATOR RESPONSIBILITIES 38](#__RefHeading___Toc107045862)

[18.0 REFERENCES 39](#__RefHeading___Toc107045863)

[APPENDIX 1: STUDY ACTIVITIES CHART 41](#__RefHeading___Toc107045864)

[APPENDIX 2: DIRECTIONS FOR USE OF STUDY GEL 42](#__RefHeading___Toc107045865)

# STUDY SUMMARY

# **Randomized Controlled Trial of Cellulose Sulfate Gel and HIV in Nigeria**

**Study 9757**

**Design:** Phase 3, multi-center, randomized, placebo-controlled trial to determine the effectiveness and safety of the 6% Cellulose Sulfate (CS) vaginal gel for the prevention of HIV infection.

**Population:** 2,160 HIV antibody negative participants:

1,080 participants using CS gel

1,080 participants using placebo gel

**Study Duration:** 12 months of participant recruitment; 12 months of product use for each participant; 26 total months in the field including screening and close-down.

**Primary Objective:** Determine the effectiveness of CS gel in preventing male-to-female vaginal transmission of HIV among HIV sero-negative women at high risk of HIV infection.

**Primary Endpoint:** Incidence of HIV-1 and HIV-2 infection as determined by detection of HIV antibodies from oral mucosal transudate (OMT) specimens.

**Secondary Objective** Determine the effectiveness of CS gel in preventing male-to-female transmission of gonorrhea and Chlamydia among women at high risk of sexually transmitted infections.

**Secondary Endpoint:** Incidence of genital gonorrhea or Chlamydia as determined by DNA probe technology from self-administered vaginal swabs.

**Study Sites:**  Port Harcourt, Nigeria

Lagos, Nigeria

# LIST OF ABBREVIATIONS AND ACRONYMS

AE Adverse Event

AER Adverse Event Report

AIDS acquired immunodeficiency syndrome

API active pharmaceutical ingredient

CS cellulose sulfate

CT *Chlamydia trachomatis*

DCF data collection form

DMC Data Monitoring Committee

DNA deoxyribonucleic acid

ELISA enzyme-linked immunosorbent assay

FDA US Food and Drug Administration

FHI Family Health International

GC *Neisseria gonorrhoeae*

GCP Good Clinical Practice

GLP Good Laboratory Practice

GMP Good Manufacturing Practice

HIV human immunodeficiency virus

HPTN HIV Prevention Trials Network

ICH International Conference on Harmonization

ID identification number

IND Investigational New Drug

IRB Institutional Review Board

KOH potassium hydroxide

Ml milliliter

Mg milligram

NIH National Institutes of Health

N-9 nonoxynol-9

OMT oral mucosal transudate

PCR Polymerase Chain Reaction

PHSC Protection of Human Subjects Committee

MIC minimum inhibitory concentration

MTT (3-(4, 5-dimethylthiazolyl-2)-2, 5-diphenyltetrazolium bromide)

NAFDAC National Agency for Food and Drug Administration and Control

RPR rapid plasma reagin

SAE Serious Adverse Event

SDA strand displacement assay

SEM scanning electron microscope

SOP standard operating procedure

STI sexually transmitted infections

TPHA *Treponema pallidum* Hemagglutination Assay

UNAID Joint United Nations Programme on HIV/AIDS

WHO World Health Organization

# 1.0 INTRODUCTION

## 1.1 Background

The Joint United Nations Programme on HIV/AIDS (UNAIDS) estimates that more than 40 million humans are infected with HIV and 5 million newly infected in 2001. AIDS resulting from HIV infection has caused the death of over 20 million people.^1^ Heterosexual contact accounts for more than 70% of all HIV-1 infections worldwide,^1^ and this mode of transmission is increasing in the United States. It is now the predominant route of infection in American women.^2^

There is a clear need for new technologies to prevent the sexual transmission of HIV. Despite years of effort, an effective HIV-1 vaccine remains elusive.^3^ Correct and consistent male condom use has been shown to prevent HIV-1 transmission,^4^ but women are often unable to negotiate the use of condoms by their male partners.^5-7^ The female condom has recently been marketed as an alternative barrier method,^7^ but use of this device requires a certain level of skill, and at least the consent of the male partner. Furthermore, the efficacy of the female condom for sexually transmitted infection (STI) prevention is unknown. Cost and limited access to the female condom are also a barrier in many countries.

Topical microbicides are products that are designed to inhibit the sexual transmission of HIV and other disease pathogens.^5,8^ Potentially, they can be applied vaginally to prevent both male-to-female and female-to-male transmission. They also offer a female-controlled prophylactic option in cases in which male condom use cannot be negotiated. Marketed chemical spermicides, which show some activity against STI pathogens in vitro, have been evaluated as topical microbicides. However, no clinical studies have yet demonstrated that these products can prevent HIV infection, and spermicides with N-9 have been shown to cause mucosal erosion and ulceration, which may increase the risk of HIV transmission.^9,10^

A vaginal microbicide effective against HIV and other STIs would be an important addition to the current preventive methods. Many of the viral STIs, such as herpes and HIV, have no cure and prevention is the only strategy for controlling the epidemics associated with STIs. Newer products have been formulated, shown promise in pre-clinical studies, and have demonstrated safety profiles in Phase 1 studies that will now permit testing for effectiveness.

## 1.2 Preclinical Studies

Sodium cellulose sulfate (CS) is a non-cytotoxic antifertility agent that exhibits antimicrobial activity *in vitro* against sexually transmitted pathogens. A gel containing 6% CS has been shown (in decreasing order of efficiency) to stimulate acrosomal loss, inhibit hyaluronidase, and impede sperm penetration into cervical mucus *in vitro.* The clinical formulation has been shown to be contraceptive in rabbits at 1 mg/ml, when the sperm and product are pre-mixed prior to vaginal inoculation, and at 50 mg/ml using vaginal application of the formulation prior to sperm introduction.^11^

The antimicrobial activity of CS has been evaluated by exposing laboratory strains, and in some cases clinical isolates, of viruses, protozoa, bacteria, and yeast to increasing doses of the drug and attempting to culture them in appropriate media. Complete inhibition of growth was observed for HIV, HSV-1, HSV-2 and HPV at concentrations less than 200 micrograms per ml. Fifty percent inhibition of N. gonorrhoeae, C. trachomatis, E. coli, G. vaginalis, T. vaginalis, C. albicans, S. aureus, and P. aeruginosa was seen at concentrations below 12 mg/ml. In addition, in vitro studies have shown that CS does not inhibit the proliferation of lactobacillus strains.^11^

Animal studies suggest that CS gel is minimally irritating to the vaginal epithelium. Vaginal irritation studies showed mild irritation in rabbits and no irritation in rats after 14 days of exposure.^11^

CS has been shown to have anticoagulant effects *in vitro* but not in human or animal studies. No changes in activated partial thromboplastin times or platelet counts were seen in the above-mentioned Phase 1 vaginal study or 14-day rabbit vaginal irritation study.^11,12^

## 1.3 Completed Clinical Studies

The hyaluronidase inhibition and contraceptive properties of CS have been known for more than 40 years. Clinical trials performed before 1973 outside the United States with a suppository containing only CS, with a lower molecular weight than the current clinical formulation, showed a high degree of contraceptive efficacy.^13^ Presently, a vaginal contraceptive suppository that contains 100 mg CS and 230 mg nonoxynol-9 (N-9) is marketed in Germany under the name of A-Gen 53. Clinical studies with this product report a high degree of safety with a slight burning sensation being the only reported side effect.^14^

In a recently completed Phase 1 study, healthy women who applied either 2.5 ml (150 mg) or 5.0 ml (300 mg) of 6% CS gel to the vagina on six consecutive days experienced less irritation than women who applied either an inactive control gel (K-Y® Jelly) or an active control gel containing N-9 (Conceptrol®). For example, colposcopic findings related to product use were observed at follow-up among one of 12 women who applied 2.5 ml of CS gel and among two of 11 women who applied 5.0 ml of CS gel, whereas such findings were observed among three of 12 women who applied the inactive control gel and among six of 12 women who applied the active control gel. Further, colposcopic findings involving disruption of the epithelium or blood vessels were observed only among women who applied the control gels.^12^

Similar results were observed in a Phase 1 study conducted among healthy men who applied 6% CS gel to the penis on seven consecutive days: irritation was observed in one of 24 men who applied 6% CS gel and three of 12 men who applied an N-9 gel.^15^

A recent CS safety study conducted by FHI in Cameroon assessed the use of CS four times per day on epithelial disruption. It was important to evaluate the safety of CS at a high frequency of use in sexually active but low-risk women before attempting a Phase 3 study in women at high-risk for HIV infection. This Phase 1 study had 54 participants that provided information for the evaluation of CS gel for safety. The CS gel group and the K-Y**®** Jelly groups were comparable at randomization and there were no apparent differences in compliance or sexual behavior during the study.

The data show that CS gel was as well tolerated as K-Y Jelly. The number of epithelial disruptions was the same in the CS gel group and the K-Y Jelly group over the 14 days of product use. Complaints of fatigue, genital itching, and abdominal pain were similar in both groups. The AEs were minor and no participant withdrew herself from the study because of symptoms. There were no serious related AEs during the study.

It was concluded that CS gel is safe and well tolerated when used up to four times per day by sexually active women.^11^

## 1.4 Ongoing and Planned Clinical Studies

Building on the initial Phase 1 studies described above, CONRAD is collaborating with the World Health Organization, the HIV Prevention Trials Network of NIH (HPTN), and FHI to implement expanded safety and acceptability studies of CS gel. The basic design of ongoing studies is shown below:

*HPTN study (HPTN 049) – is ongoing in HIV-infected women:*

|  | **Study Cohort** | **n, CS Gel** | **n, K-Y® Jelly** | **Frequency of Use** | **Duration of use** |
| --- | --- | --- | --- | --- | --- |
|  | 1: Sexually Abstinent | 12 | 12 | Once Daily | 14 days |
|  | 2: Sexually Abstinent | 12 | 12 | Twice Daily | 14 days |
|  | 3: Sexually Active | 12 | 12 | Once Daily | 14 days |
|  | 4: Sexually Active | 12 | 12 | Twice Daily | 14 days |

*CONRAD (protocol A01-068) – is ongoing in the US and Dominican Republic:*

|  | **Study Cohort** | **n, CS Gel** | **n, K-Y® Jelly** | **Frequency of Use** | **Duration of use** |
| --- | --- | --- | --- | --- | --- |
|  | 1: Sexually Abstinent | 15 | 15 | Twice Daily | 14 days |
|  | 2: Sexually Active | 15 | 15 | Twice Daily | 14 days |

The planned studies with CS include: a penile study among HIV positive men; a study in Zimbabwe where women will be randomized to one of three arms: diaphragm with CS; diaphragm with KY Jelly and CS alone; a contraceptive trial in the US.

## 1.5 Rationale

Efforts to develop anti-HIV vaccines continue; however, other key means to minimize the spread of HIV must be employed. Male condom use is effective in preventing infection by HIV and other sexually transmitted infections but social and cultural mores in certain populations may deter this form of transmission control [16]. Therefore, there is a need for additional strategies to prevent the spread of HIV, particularly as it applies to women who are at high risk for HIV acquisition.

Topical intravaginal microbicides may offer women a means to control sexual transmission of HIV in settings where they have limited ability to have their male partners use condoms. Based on the pre-clinical and clinical data, CS gel appears to be a promising candidate. We will conduct a Phase 3 study to evaluate the effectiveness of CS gel in preventing vaginal HIV transmission among young, sexually active HIV sero-negative women at high-risk for acquiring HIV infection.

# 2.0 STUDY OBJECTIVES

## 2.1 Primary Objective

Determine the effectiveness of 6% CS gel in preventing male-to-female vaginal transmission of HIV among HIV sero-negative women at high risk of HIV infection.

## 2.2 Secondary Objectives

Determine the effectiveness of 6% CS gel in preventing male-to-female transmission of gonorrhea and Chlamydia among women at high risk of STIs.

# 3.0 TRIAL DESIGN

## 3.1 Primary Endpoint

Combined incidence of HIV-1 and HIV-2 infection as determined by 1) detection of HIV antibodies from OMT specimens and confirmed by an ELISA or a Western blot from a finger prick specimen at a study follow-up visit, or 2) by presence of HIV DNA material detected by a PCR test at the final follow up visit.

## 3.2 Secondary Endpoints

Incidence of genital gonorrhea or Chlamydia as determined by DNA amplification/probe technology from self-administered vaginal swabs.

## 3.3 Study Design

The study will be conducted in compliance with the protocol, FHI SOPs, Good Clinical Practice (ICH/GCP), and the applicable regulatory requirements of the FDA and the regulatory agencies of the site country.

This is a Phase 3 multi-center, randomized, placebo-controlled trial to assess the effectiveness of CS gel in preventing male-to-female vaginal transmission of HIV among HIV sero-negative women at high risk of HIV infection. Eligible participants who are HIV negative, at risk of becoming infected, and are willing to use a vaginal microbicide each time they have intercourse throughout 12 months of study participation will be recruited in Port Harcourt and Lagos, Nigeria. The enrollment phase will last until 2,160 women have enrolled. It is anticipated that the enrollment will be completed within 12 months.

Enrolled participants will be randomized into either the CS or placebo arm on a 1:1 allocation ratio. A total of 2,160 participants will be enrolled: half of the participants using CS gel and the other half using placebo gel. Participants will have monthly follow up visits. At each visit they will:

- Receive additional study product
- Undergo a structured interview
- Give urine for a pregnancy test
- Give an OMT specimen for HIV test; give blood by a finger prick for the confirmation test if OMT test is reactive
- Give vaginal swab for GC and CT tests
- Receive HIV and STI risk reduction counseling

There will be a blood draw for PCR testing at the final study visit.

All women will be counseled to use condoms every time they have intercourse throughout the study to minimize HIV/STI transmission. Condoms will be provided to all participants as part of the study. They may use any additional non-vaginal contraceptive of their choice, although none will be provided as part of this study.

The HIV status at screening and enrollment will be determined by reactive OraQuick result confirmed by ELISA. Only discordant OraQuick and ELISA results will be confirmed by Western blot at screening and enrollment. Participants who have a reactive OraQuick result at screening or enrollment will not be admitted to the study regardless of their confirmatory results.

All participants who are confirmed HIV-positive at screening or enrollment or acquire the HIV infection during the study will be counseled and referred to the appropriate medical care in accordance with the local regulations. We will not provide treatment for HIV. Participants who acquire HIV during the study will not be discontinued from follow-up.

We will confirm all reactive OraQuick results that occur during the study (after enrollment into the study) by both ELISA and Western blot. Additionally we will perform PCR using blood specimens collected at the enrollment visit to verify that participants whose OraQuick results turn reactive within their first three months of participation in the study were HIV negative at the time of enrollment. We also will perform PCR on all blood specimens collected at the final study visit and not found to be positive by Elisa and Western blot to ensure that we detect as many early seroconversions as possible.

## 3.4 Measures to Minimize Bias

### 3.4.1 Randomization

A Randomization Manager who is not otherwise involved with the study will develop the allocation sequence using a computer random number generator and randomly varied permuted-blocks. The two arms of the study (CS gel and placebo gel) will each be divided into three randomization groups to maintain blindness. Each group will be color-coded. There will be a total of six color-coded randomization groups in the study. Participants will be randomized to one of the six groups only after they have qualified for the study and signed the consent form. Randomization will be stratified by study site.

### 3.4.2 Allocation concealment

Group assignments will be concealed in sequentially numbered, sealed opaque envelopes. Trained staff will open the envelopes only after participants are properly enrolled. The randomization envelopes will be maintained in a secure office. They will not be available to the study counselors until the immediate moment of randomization. Each randomization envelope will be used only once.

### 3.4.3 Blinding

Our goal is to blind: 1) participants, 2) site staff, and 3) FHI staff throughout the conduct of the study. However, since the appearance of CS and placebo gels is not identical, we cannot be completely assured that an unblinding will not occur. All measures will be taken to prevent an unplanned unblinding:

- the CS and placebo gels will be identical in packaging, labeling, and applicators
- the opaque applicators will conceal the different appearance of the study gels
- the two arms of the study will each be divided into three groups. Therefore, there will be a total of six groups (three CS groups and three placebo groups). Each group will be color-coded and participants and staff will not know which groups are CS or placebo
- the research staff will use the participant’s actual assigned gel and/or empty applicators for demonstration purposes. All demonstrations will be independent so no participant will see any other participant's gel
- FHI data analysts will be blinded until the final primary analysis programs have been verified independently using blinded groups
- All attempts will be made to keep the FHI clinicians blinded until the final analysis programs have been verified

Should an unplanned unblinding occur at the site, the Investigator will immediately notify FHI.

### 3.4.4. Randomization codes and decoding procedure

The FHI Randomization Manager will maintain the trial treatment randomization codes. The Manager will provide the code to the DMC during meetings to review the interim data. The codes will be made available to Dr. David Grimes, FHI Chief Medical Officer, should the need arise to reveal the code. It is anticipated that the unblinding will occur rarely and then only in the case of an SAE in which the knowledge of study gel would affect care of the participant.

## 3.5 Duration of the Study

The screening phase will start 1 month before the enrollment phase and end 1 month before the end of the enrollment phase. Participants will be actively enrolled until 2,160 women have enrolled. It is anticipated that enrollment will last 12 months. Once enrolled, participants will actively use CS gel or placebo gel for 12 months. Participants will return for monthly follow-up visits. The study will remain open for at least 1 month after the last scheduled follow-up visit to allow follow-up of ongoing AEs and attempts to find participants that have not returned for their scheduled visits.

## 3.6 Discontinuation Criteria

### 3.6.1 Individual participants

**Discontinuation of the study**

A participant may be discontinued early from the study for any of the following reasons:

•        She considers it to be in her best interest or for personal reasons

•        A physician considers it to be in the participant's best interest because of safety reasons and/or for the well-being of the participant, and

Reasons for discontinuation from the study will be recorded on the appropriate DCF. **When early discontinuation from the study occurs, every reasonable effort will be made to assess information relevant to** **the endpoints at the time of discontinuation**. This would include attempts to obtain specimens for HIV, GC and CT. Withdrawn participants will not be replaced.

**Study product interruption**

Treatment with the study product may be interrupted temporarily or permanently for any of the following reasons:

•        The participant ran out of supplies for whatever reason (e.g., she missed a visit); in this case the date of interruption is the date of her last product use

•        An AE

- The Investigator decides to withdraw the study product in the interests of the safety and well being of the participant
- The participant has to use another vaginal medication
- The participant considers it to be in her best interest or personal reasons

•        Pregnancy

If a woman becomes pregnant during the study she will interrupt the use of the study product. She will be encouraged to stay in the study and complete the follow up: she will be tested for HIV, gonorrhea and Chlamydia each month. She will be discontinued from the study if she wants. However we will collect data on the outcome of her pregnancy as soon as this information is available, even after the end of the study

Whenever the study product is interrupted, the information will be noted on the follow-up DCF including the actual date when the study product was stopped.  If possible, the follow-up should be continued until the participant completes the study or resumes study product.  **If possible, participants will be tested for HIV, GC and CT so that the endpoints are determined at the time of product interruption.**

Participants who have had study drug interruption may resume study product use and will be given the study product to which they were originally randomized (unless the Investigator determines for safety reasons that the participant should not continue use of the gel).  **At the time of product resumption the participant will be tested for HIV, GC and CT so that status of the endpoints is determined**.

### 3.6.2 Study discontinuation

The study will continue until the planned end of the study, or until such time as:

- FHI decides to discontinue the study. FHI may discontinue a site/Investigator due to noncompliance with protocol or regulations
- CONRAD decides to suspend or discontinue testing, evaluation, or development of CS
- The IRB decides to discontinue the study
- The US FDA terminates the study (withdraws approval of the IND)
- The National Agency for Food and Drug Administration and Control of Nigeria (NAFDAC) terminates the study

## 3.7 Study Product Accountability

FHI will be responsible for controlling the shipment of the investigational drug. The study sites will receive a supply of study gels sufficient for the anticipated number of study participants. All supplies must be stored at room temperature (15C-30C) in a limited access area that is securely locked.

The study site will also receive male lubricated (not nonoxynol-9 lubricant) latex condoms to dispense for use with the study gels.

For purposes of inventory accountability, only appropriate site personnel can distribute supplies of the study gels; gels cannot be given to any person not enrolled in this trial.

The study site is required to maintain records of the disposition of each unit of study product received and dispensed, including dates and quantities of distribution to participants, by participant ID number. All unused supplies of the study product must be returned at the end of the study, or disposed of at the site in a manner specified by CONRAD. The study site will not be required to record the disposition of the male latex condoms provided for the study.

## 3.8 Data Directly Recorded on DCFs

All DCFs used in the field for this study will have data that are directly recorded on them and will be considered source documents.  Other source documents include, but are not be limited to, staff journals, medical notes, screening and enrollment logs, laboratory results, gel dispensing records, consent forms, and reimbursement logs.

# 4.0 SELECTION AND WITHDRAWAL OF PARTICIPANTS

The target population is sexually active women who are at high risk for acquiring HIV. High risk for the purposes of this study includes having intercourse, on average, three times per week and having more than one sexual partner in the last 3 months.

## 4.1 Eligibility Criteria

To be eligible for inclusion in this study, a woman must:

- Be willing and able to give informed consent
- Be at least 18 years old and not more than 35 years old
- Average three vaginal coital acts per week with a male partner
- Have had more than one male sexual partner in the last 3 months
- Be willing to use study product as directed
- Be willing to adhere to follow-up schedule
- Be willing to participate in the study for 12 months
- Be willing to report self-medication during study participation
- Be willing to give urine for pregnancy testing, self-administered vaginal swabs for GC/CT testing, OMT for HIV monthly, finger prick for HIV confirmation if required, blood draw for syphilis and HIV at baseline, and for HIV - at the final visit
- Be willing to not use a spermicide, other vaginal contraceptive, or vaginal lubricant during the study
- Be at least 3 months since end of the last pregnancy
- Not have a history of adverse reaction to the study products, including latex
- Not be pregnant or desire a pregnancy during the 12 months of participation
- Not be an injection drug user
- Not have a gynecological abnormality that may have an impact on the safety and/or response to the study gel according to the Investigator
- Not be HIV positive as diagnosed by OraQuick® rapid test
- Not participate in any other microbicide research
- Not being discontinued from the CS study previously
- Not have any condition (social or medical) which in the opinion of the investigator would make study participation unsafe or complicate data interpretation

If a participant has a gonorrhea and/or Chlamydial infection at screening, she will be treated as soon as possible and she may be enrolled either with untreated infection or while being treated for infection. Eligibility for enrollment is strictly based on HIV sero status. Once a participant has had a negative laboratory test for gonorrhea or Chlamydia, a vaginal specimen will be tested every month thereafter to assess for re-infection. If a participant is HIV-positive at screening or enrollment, she will be counseled and referred appropriately according to the local regulations. She will not be eligible for the study. A participant with reactive OraQuick result will not be eligible for the study regardless of her HIV confirmatory results.

## 4.2 Completion

A participant will be considered to have completed the study after she has completed 12 months of using CS or placebo gels, and after her final set of data has been collected and entered on the appropriate DCFs.

## 4.3 Loss to Follow-up

If a participant fails to appear for a scheduled visit, at least three attempts to contact her will be made over the subsequent 30 days. These attempts will be documented in the participant’s study file. Her file will remain open until study closeout.

If the participant does not return to the study before the study is closed, the Final Status Form will be completed at the time of study closeout. The form will indicate that the participant was lost to follow-up. The “lost to follow-up” designation cannot be made for any participant until the closing date of the study.

# 5.0 STUDY PRODUCT AND TREATMENTS

## 5.1 CS Gel

Six percent CS gel contains 60 mg of the active ingredient, sodium cellulose sulfate, per gram of gel. Each 3.5 ml application of 6% CS gel contains 231 mg of the active ingredient. The vehicle gel contains 5% sorbitol and 5%glycerin ashumectants, 0.25% carbopol as a thickener, 1% benzyl alcohol as preservative and water. The inactive ingredients are United States Pharmacopeia/ National Formulary grade materials. Addition of the active ingredient to the vehicle at a 6% concentration produces a thick gel that should be stored at room temperature. The pH of 6% CS gel is 7.5 (neutral).

Clinical supplies of CS 6% gel have been manufactured and packaged under Good Manufacturing Practice (GMP) conditions for this study in disposable, single-use over-wrapped applicators. Gel and delivery volume of the loaded applicators is being monitored for stability for the duration of the clinical studies.

CS 6% gel will be administered in a 3.5ml dose via an applicator. CS gel is most effective when administered immediately before intercourse. Participants should insert CS gel into their vagina before each and every sex act. However, if intercourse does not occur within one hour after application, they should reapply the gel. Participants will be instructed to return for a monthly study visit, which at this time will receive a supply of assigned study product. Please see Appendix 2 for directions of product use.

## 5.2 Placebo Gel

The placebo gel is formulated to minimize any possible effects — negative or positive — on study endpoints. It is isotonic to avoid epithelial cell swelling or dehydration. It is formulated at a pH of 4.4 but has minimal buffering capacity. When mixed with an equal volume of semen, the placebo gel induced only a trivial decrease in semen pH (from 7.8 to 7.7).

The placebo gel contains hydroxyethylcellulose as a gelling agent, and its viscosity is comparable to that of K-Y Jelly Personal Lubricant. Hydroxyethylcellulose does not have anti-HIV properties. The gel also contains sorbic acid as a preservative. Sorbic acid has no anti-HIV activity and is readily metabolized by human cells.

The placebo gel will be administered in a 3.5ml dose via an applicator identically to CS application.

## 5.3 Gel Labeling

Both study gels will be pre-filled in identical opaque applicators. Each applicator will be individually wrapped and labeled with a Caution statement that this is an investigational drug. Boxes of 10 or 20 individually wrapped applicators will be packaged for each participant. Each box will be labeled with the Caution statement and color code, and have the protocol number indicated.

Both study gels will be provided by CONRAD. The site will be provided with enough supplies for participants. The site must maintain complete records of all gel supplies received and subsequently dispensed to and returned by study participants.

All unused applicators should be returned to the clinic, inventoried, and then destroyed in accordance with conditions specified in the Transfer of Obligations signed between CONRAD and FHI.

The study site will be supplied with a single brand and type of lubricated latex male condom (not containing N-9) for distribution to study participants.

## 5.4 Concomitant Treatments

All concomitant medications used during the study will be recorded on applicable study DCFs. Vaginal medications should not be used concurrently with the study gel. Gel use will be interrupted if a participant needs to use a vaginal medication and the procedures for product interruption will be followed.

## 5.5 Monitoring of Product Compliance

We will assess gel and condom use by interview.  We will ask the number of vaginal sex acts the woman had in the 7 days prior to the interview, and among those acts, the number of gels used, the number protected by condoms, the number without condoms or gel, and the number with condoms and gel use together.

# 6.0 EFFECTIVENESS ASSESSMENT

## 6.1 Effectiveness Parameters

### 6.1.1 HIV effectiveness

The effectiveness of CS gel against HIV (1 and 2) will be measured by comparing the incidence of HIV antibody detection in the CS group with that in the placebo group.

An incident HIV infection is defined as follows:

- The presence of HIV antibodies by OraQuick® rapid test, confirmed by ELISA or Western blot from serum collected by a finger prick at a follow-up visit, or the presence of HIV DNA material detected by a PCR test at the final visit
- The absence of HIV antibodies by OraQuick or the presence of an unconfirmed reactive OraQuick result on a sample collected at the previous visit
- The absence of an HIV-positive PCR result at the enrollment visit

### 6.1.2 GC or CT effectiveness

The effectiveness of CS gel against GC or CT will be measured by comparing the incidence of organism detection by SDA from self-administered vaginal swabs in the CS group with that in the placebo group.

An incident GC or CT infection will be defined as follows:

- The presence of a positive SDA test at a follow-up visit, and
- The absence of a positive SDA test on a sample collected at the previous visit

## 6.2 Methods and Timing of Effectiveness Parameters

### 6.2.1 HIV effectiveness

OMT specimens will be tested for HIV-1 and HIV-2 antibodies at screening, enrollment, and then monthly throughout follow-up using OraQuick. OraQuick HIV-1 and HIV-2 is a rapid test device that can detect antibodies to HIV in oral mucosal transudate. OraSure Technologies, Inc makes OraQuick.

The OraQuick results will be available 20 minutes after collection. If the OraQuick test is positive **during the study follow up**, the participant will be told that the test results are preliminary and a finger prick specimen will be collected on filter paper and tested by both ELISA and a Western blot for confirmation of HIV infection. All participants with negative or indeterminate Western blot results and positive Elisa results will undergo re-testing in one month.

We will perform PCR using blood specimens collected at the enrollment visit to verify that participants whose OraQuick results turn reactive within their first three months of participation in the study were HIV negative at the time of enrollment. Additionally we will perform PCR on all blood specimens collected at the final study visit and not found to be positive by Elisa and Western blot to ensure that we detect as many early seroconversions as possible. The enrollment blood specimens from all participants with positive PCR results at the final study visit will be re-tested by PCR. Those participants who are found to have been HIV positive at enrollment will be excluded from the effectiveness analyses. All other participants with positive PCR results at the final study visit will be considered to have reached the study endpoint for the purpose of the analysis.

It is recognized that some combinations of positive, negative, indeterminate, or missing OMT, Elisa, Western blot, and PCR results will arise that require individual consideration or additional testing to determine whether a study event has occurred. All such patterns of results will be evaluated in a blind review of the data and without knowledge of gel group, pregnancy, adverse event, or other STI outcomes. Decisions regarding why each pattern was included or excluded from the set of study infections will be documented in the final study report.

Blood draw will be collected for PCR testing at the enrollment and final study visits and shipped to the Institute of Tropical Medicine (ITM) in Belgium. ITM will use AmpliScreen HIV-1 test (Roche Diagnostics) to perform PCR testing.

### 6.2.2 GC and CT effectiveness

Self-administered vaginal swabs will be collected at screening, enrollment, and then at monthly follow-up visits throughout the study. The specimens will be tested for GC and CT using SDA. SDA is a molecular system that uses amplification and DNA probes to provide excellent sensitivity and specificity for diagnosing GC and CT. Becton, Dickinson and Company makes the SDA equipment.

There will be two swabs self-collected by each participant at each of the study visits. One swab will be tested for CT and GC by the central labs in Lagos and Port Harcourt. The second swab will be shipped to ITM in Belgium for quality control purposes.

# 7.0 ADVERSE EVENTS AND REPORTING REQUIREMENTS

## 7.1 Adverse Events

An Adverse Event (AE) is any unfavorable and unintended sign, symptom, or disease temporally associated with the use of a medicinal product, whether or not considered related to the medicinal product. Pre-existing events, which increase in frequency or severity or change in nature during or because of use of a drug in human clinical trials, will also be considered as AE. AE’s may also include pre‑ or post‑treatment complications that occur as a result of protocol-mandated procedures (e.g., invasive procedures such as biopsies).

Any medical condition or clinically significant laboratory abnormality with an onset date before the first date of study drug administration is considered to be pre-existing, and should be documented on the DCF noting that it is pre-existing.

Any AE (i.e., a new event or an exacerbation of a pre-existing condition) with an onset date after study product administration up to the last day on study (including the follow-up) should be recorded as an AE on the appropriate DCF. If the participant experiences any abnormal genital bleeding (other than menstrual), an Intermenstrual Bleeding (IMB) Form will be completed in addition to the AE Form.

Study participants will be provided with contact information and instructions to contact the Investigator or designee to report any AE they may experience, except for life-threatening events, for which they will be instructed to seek immediate emergency care. Based on the results of the Investigator’s assessment, the Investigator will recommend either continuation or discontinuation of product use. The Investigator also may prescribe or recommend the use of medications or other preparations to treat the AE.

Where feasible and medically appropriate, participants will be encouraged to seek medical care where the site study clinician is based, and to request that the clinician be contacted upon their arrival. With appropriate permission of the participant, records from all non-study medical providers related to AEs will be obtained and required data elements will be recorded on study DCFs. All participants reporting an AE will be followed clinically, until the AE resolves (returns to baseline) or stabilizes.

The Investigator must determine the severity of the AE and document on the appropriate DCF. Each AE that the participant is aware of should be graded for severity using the following scale:

- **Very slight:** participant was aware of the AE on and off
- **Mild:** participant was aware of the AE all of the time, but was still able to do all activities
- **Moderate:** the participant had to discontinue some activities due to the AE
- **Severe:** the participant was incapacitated by the AE and unable to perform normal activities

An AE **does not** include:

- Medical or surgical procedures (e.g. surgery, endoscopy, tooth extraction, transfusion); the condition that leads to the procedure is an AE.
- Pre-existing diseases or conditions present or detected prior to start of study drug administration that do not worsen.
- Situations where an untoward medical occurrence has not occurred (e.g. hospitalization for elective surgery, social and/or convenience admissions).
- Overdose of either study drug or concomitant medication without any signs or symptoms unless the participant is hospitalized for observation.

## 7.2 Relationship of AE to study product

The Investigator must determine the relationship of the AE to the product under investigation and document on the appropriate DCF. For each AE, an assessment of the relatedness to the test agent should be made using the following scale:

- **Unrelated**: Onset of the AE had no reasonable temporal relationship to administration of the study product or a causal relationship to administration of the study product is biologically implausible or the event is attributed to an alternative etiology.
- **Possibly Related**: Onset of the AE has a reasonable temporal relationship to study product administration and a causal relationship is not biologically implausible.
- **Probably Related**: Onset of the AE has a strong temporal relationship to administration of the study product that cannot be explained by the participant’s clinical state or other factors and a causal relationship is not biologically implausible.
- **Definitely Related**: Onset of the AE shows a distinct temporal relationship to administration of the study product that cannot be explained by the participant’s clinical state or other factors or the AE occurs on rechallenge or the AE is a known reaction to the product or chemical group or can be predicted by the product’s pharmacology.

These criteria in addition to good clinical judgment should be used as a guide for determining the causal assessment. If it is felt that the event is not related to study drug therapy, then an alternative explanation should be provided.

## 7.3 Reporting AEs

Study site staff will document on the appropriate study DCF all AEs reported by or observed in enrolled study participants regardless of severity and presumed relationship to study product. The study site clinician/Investigator must provide on this form information on symptoms, time of onset, severity, frequency, product-relatedness, action(s) taken, and participant outcome. The FHI’s study clinician may request additional information from the site if it is needed to evaluate the AE. Site staff will report information on all AEs to the local IRB in accordance with US Federal regulations and local IRB requirements.

## 7.4 Serious Adverse Events

A **Serious Adverse Event** (SAE) for a drug or biologic product or medical procedure is defined as any experience that suggests a significant hazard, contraindication, side effect, or precaution. A SAE includes any adverse experience that results in any of the following outcomes:

- Death
- A life-threatening adverse drug experience
- Inpatient hospitalization or prolongation of existing hospitalization
- A persistent or significant disability/incapacity
- A congenital anomaly/birth, or
- An important medical event that, based on medical judgment, may jeopardize the patient or subject and may require intervention to prevent one of the outcomes listed above.

Examples of such events are:

- Intensive treatment in an emergency room or at home for allergic bronchospasm
- Blood dyscrasias or convulsions that do not result in hospitalization
- Development of drug dependency or drug abuse

**Clarification of SAEs:**

- Death is an outcome of an AE, and not an AE in itself.
- All deaths, regardless of cause, must be reported for participants on study and for deaths occurring within 30 days of last study product use or within 30 days of last study evaluation, whichever is longer.
- “Occurring at any dose” does not imply that the participant is using study product at the time of the event. Product use may have been interrupted temporarily prior to the onset of the SAE, but may have contributed to the event.
- “Life-threatening” means that the participant was at immediate risk of death from the event as it occurred. This does not include an event that might have led to death, if it had occurred with greater severity.
- Complications that occur during hospitalizations are AEs. If a complication prolongs hospitalization, it is a SAE.
- “Inpatient hospitalization” means the participant has been formally admitted to a hospital for medical reasons, for any length of time. This may or may not be overnight. It does not include presentation and care within an emergency department unless the participant is “admitted”.
- The Investigator should attempt to establish a diagnosis of the event based on signs, symptoms and/or other clinical information. In such cases, the diagnosis should be documented as the AE and/or SAE and not the individual signs/symptoms.

## 7.5 Reporting SAEs

All SAEs should be reported to FHI **as soon as possible** after the SAE is identified by site personnel. FHI strongly recommends that site personnel report SAEs within 24 hours of the study site becoming aware of the problem. The Investigator should complete a FHI SAE Report Form and coversheet and fax it to:

Janet Robinson

Director, RA/QA

Family Health International

Telephone: 919-544-7040, ext. 351

Fax: 919-544-1380

Email: jrobinson@fhi.org

In cases in which a SAE Report Form cannot be faxed within 24 hours, the Investigator may report the SAE via telephone or e-mail; however, a SAE Report Form must be completed as soon as possible after the informal report. Based on the preliminary information, the FHI Study Clinician will complete an interim SAE Report Form and forward it to the PHSC Representative for processing. The study clinician will make sure to update this information as soon as he/she receives a complete SAE Report Form from the Investigator.

If the SAE **is unexpected, and possibly, probably or definitely related to the use of an investigational product**, the FHI Project Leader/Study Clinician will alert CONRAD within 5 calendar days of receipt of the SAE Report Form that an SAE has occurred that may be reportable to the regulatory agency. If the SAE is also **fatal or life-threatening**, the FHI Project Leader/Study Clinician will report it to CONRAD immediately.

The FHI Project Leader/Study Clinician will process and report all SAEs to CONRAD and PHSC in accordance with the FHI’s SOP#301 “Processing of AE Reports for Clinical Studies”.

The Investigator will be responsible for proper reporting of all SAEs to the local IRB and FHI as specified in the protocol.

## 7.6 Social Risk Events

No social risk events are expected from participation in this study. However, if the Investigator learns of a social risk event, he will report the event on the Social Risk Event Form and fax or mail it to:

Janet Robinson

Director, RA/QA

Family Health International

P.O. Box 13950

Research Triangle Park, NC 27709,USA

Telephone: 919-544-7040 ext.351

Fax: 919-544-1380

# 8.0 STUDY VISIT SUMMARY

Participants will make visits to a study clinic for one screening, one enrollment, and 12 monthly follow-up visits. See Appendix 1 for a table of study procedures and timing of activities.

## 8.1 Screening Visit

Screening visit will take place in the clinic. The study, including potential risks and benefits of participation, will be explained to prospective participants during screening. This will be a general overview of the study purpose and procedures. Each woman will be asked to sign (or provide other mark) a consent form for the screening procedures, and will be given a signed copy of the consent form to take home. If the participant is illiterate, a witness will be present at the time of the study explanation and the consent process, and will witness the signature or mark of the participant.

All volunteers will be assigned an ID number and evaluated to determine their eligibility according to the participation eligibility criteria.

The following will be done:

- An interview with the woman to make sure she understands the study schedule and is willing to comply with study requirements
- Consent process for the screening procedures
- Pre-test HIV counseling
- A urine pregnancy test
- OMT specimen taken for HIV testing; a finger prick specimen will be taken for the confirmation test if OMT test is reactive
- Post-test HIV counseling
- Pelvic examination
- A vaginal swab for GC and CT SDA testing
- STI risk reduction counseling

The women will be asked to return in 4 weeks for the enrollment visit. The participants with positive HIV and/or STI results will be contacted as soon as the results become available.

If the OraQuick test is reactive at screening, the participant will be told that the test results are preliminary and a finger prick specimen will be collected on filter paper and tested by ELISA. A Western blot will be done for confirmation of HIV infection only if the results of ELISA and OraQuick are discordant.

If a woman has a reactive OraQuick test at screening that is confirmed by ELISA or Western blot, she is not eligible for the study. But she may speak with a study counselor. A participant diagnosed with HIV will not be treated for HIV. The study staff will refer her to appropriate local resources to receive additional support and care. A participant with a reactive OraQuick result at screening that is not confirmed by ELISA or Western blot, is also ineligible for the study. However we may ask her to come back voluntarily for more confirmatory tests if needed that will be provided for free.

Women with any curable STI diagnosed at the screening will be treated and may be enrolled in the study. Whenever possible, single dose directly observable treatments will be used for all treatments in this study. The STI treatments approved by the local Drug Board and Ministry of Health or CDC treatment guidelines will be used.

All women will be provided with condoms, instructed in proper condom use and asked to use them during each act of sexual intercourse.

***Pelvic exam***

The following examination and specimen collection sequence will be followed:

- Inguinal lymph nodes palpated and enlargement and tenderness noted
- Vulva and perineum visually inspected for breaks in epithelial integrity, warts, or other lesions.
- Vaginal introitus inspected for discharge and odor
- A speculum lubricated with water will be inserted into the vagina
- Inspection of the vagina and cervix
- Vaginal pH measured by holding a pH strip against the lateral vaginal wall with a cotton tipped applicator and compared to the pH chart provided with the strips
- A vaginal swab from the posterior fornix placed in a test tube with a few drops of normal saline for preparation of the wet-mount (saline and potassium hydroxide)
- A vaginal swab for GC and CT
- A bimanual pelvic examination performed

## 8.2 Enrollment Visit

Enrollment visit will take place in the clinic 4 weeks after the screening. There will be a 15-day grace period allowed around (before and after) the scheduled date of enrollment.

If the OraQuick test is reactive at enrollment, the participant will be told that the test results are preliminary and a finger prick specimen will be collected on filter paper and tested by ELISA. A Western blot will be done for confirmation of HIV infection only if the results of ELISA and OraQuick are discordant.

If a woman has a reactive OraQuick test at enrollment that is confirmed by ELISA or Western blot, she is not eligible for the study. But she may speak with a study counselor. A participant diagnosed with HIV will not be treated for HIV. The study staff will refer her to appropriate local resources to receive additional support and care. A participant with a reactive OraQuick result at enrollment that is not confirmed by ELISA or Western blot, is also ineligible for the study. However we may ask her to come back voluntarily for more confirmatory tests if needed that will be provided for free.

If the woman has a non-reactive OraQuick result and meets all other eligibility criteria, she will receive a detailed explanation of study procedures, and counseled in the importance of returning for follow-up. She will be asked to sign an enrollment consent form, and will be given a signed copy to take home. If the participant cannot read, a witness will be present at the time of the study explanation and the consent process, and will witness the signature or mark of the participant. She will then be enrolled in the study and randomly assigned to one of the study groups.

***Contact information***

For each participant, clinic staff will obtain contact information. The study site will develop its own Locator Form, and determine the best way to collect this information for its own study population. In the event that a participant misses a scheduled appointment, the clinic staff will try to establish communication with the participant through all authorized possible means (e.g., telephoning if this is possible, writing to the participant and contacts, and/or visiting the participant’s home or workplace). The need to return for all scheduled follow-up visits will be emphasized to all study participants at every visit.

***Counseling***

The counselor will review study product use, and methods of HIV/STI transmission and prevention. The use of condoms with all partners, including primary sexual contacts, will be emphasized to all participants. Staff will also stress that this is a controlled trial of a product of unknown effectiveness in the prevention of HIV transmission and that condoms should be used for all sexual contacts with all partners. This information will be repeated at each follow-up counseling session for both groups.

***Laboratory testing***

At enrollment, staff will collect an OMT specimen for HIV testing and the results of the OraQuick HIV test will be known before randomization takes place. Women will be taught how to use self-administered vaginal swabs. A vaginal swab will be tested for GC and CT using SDA method. A urine pregnancy test will be conducted and pregnant women will be excluded from study participation. Blood will be drawn for syphilis and for HIV testing by PCR. Women diagnosed with syphilis or other curable STIs will be treated and may be enrolled in the study.

***Study Product***

Each participant will receive a supply of CS or placebo gels, and male latex condoms. Participants will receive supplies in quantities anticipated to last until the next visit. Staff will teach the participants how to use applicators with the gels and condoms. A participant will be instructed to return between scheduled visits to obtain more supplies if needed. A product-dispensing log will be maintained by the clinic and field staff to track distribution of the study gels. The study staff will not be required to track disposition of condoms.

***Pelvic* *examination***

A pelvic examination will only be done if the participant has symptoms or a problem that needs to be checked. If a wet mount is positive for *T. vaginalis,* clinical signs or symptoms of a yeast infection with yeast seen on a KOH wet mount, or bacterial vaginosis (Amsel criteria), the woman can be enrolled while starting treatment. Treatment will be recorded on the appropriate DCF.

## 8.3 Follow-up Visits

All follow up visits except the final one, will be conducted by outreach workers at the outreach posts. The final study visit will be conducted in the clinic.

Each participant will have a follow-up visit at a site in convenient location. Each outreach worker will be assigned to a site and will use the site as a base of operation. The outreach worker is responsible for monthly counseling, interviewing, re-supplying the participants with products, testing for HIV and pregnancy, collection of self-administered vaginal swabs for CT and GC testing, and, if necessary, home visits to ensure that each participant completes the study. The following will be done at the monthly visits:

- A questionnaire to collect information on use of gel and condoms, and any genital symptoms
- Counseling about gel and condom use
- Re-supply of gel and condoms in an amount sufficient to last until the next follow-up visit
- Pre and post test HIV counseling
- STI risk reduction counseling
- OMT specimen for HIV testing; a finger prick specimen will be taken for the confirmation test if OMT test is reactive
- A self-administered vaginal swab for GC and CT SDA testing
- A urine pregnancy test

If at any visit, the staff person or the study participant thinks that a pelvic examination is necessary or that an AE has occurred, study staff will refer the participant to a study clinic for an examination by a study nurse or a study physician.

The 12-month follow up visit (or any other visit when a participant is discontinued early from the study), will be considered a final study visit. Participants will be asked to return to the clinic for the final study visit. At this visit, in addition to the standard follow up procedures, there will be 1) a blood draw for PCR testing; 2) an Acceptability Questionnaire completed; 3) a Final Status form completed. Participants will not receive gel at the final study visit.

**HIV**

If the OraQuick test is reactive during the study follow up, the participant will be told that the test results are preliminary and a finger prick specimen will be collected on filter paper and tested by ELISA and Western blot. All participants with negative or indeterminate Western blot results and positive Elisa results will undergo re-testing in one month.

All confirmatory HIV results during the study will be delivered to the participants in the clinic as part of the post-test counseling by the trained personnel. A participant diagnosed with HIV will not be treated for HIV within the study. But she may return to the clinic and speak with a study counselor for the duration of the study. The study staff will refer her to appropriate local resources to receive additional support and care.

A participant with positive PCR result at the final study visit will be considered to have reached the study endpoint for the purpose of the analysis unless her enrollment PCR was also positive. However, we will contact and ask her to come back for a confirmatory test (ELISA or Western blot) even after her discontinuation from the study in order to provide the best medical care.

**STI**

Whenever a curable STI is diagnosed, treatment will be given to the participant and recorded on the DCF. If one of the laboratory tests is positive without clinical evidence of an STI and/or if a prescribed treatment has to be adjusted after laboratory results become available, the participant will be contacted and treated (study product can be continued).

Where possible, the participant’s partners will be referred for treatment of STI.

## 8.4 Interim Contacts and Unscheduled Visits

The participant or the Investigator may request an interim contact or an unscheduled visit at any time during the study. All interim contacts and unscheduled visits will be documented in the participants’ study records and on applicable DCFs.

Interim or unscheduled visits are distinguished from the AE-related visits in that no health related reaction, effect, or abnormality is reported. Some examples of reasons for an interim or unscheduled visit not in response to an AE are:

- To get more product or condoms
- To ask questions of study staff
- To discuss problems with study compliance
- Interim examination - examination requested but no complaints or symptom (if a pelvic exam is conducted a swab will be collected for GC and CT SDA testing and a pelvic exam DCF will be filled in)

# 9.0 STATISTICAL SUMMARY

## 9.1 Study Size Justification

With an overall study size of 2,160 participants (of which 1,080 are in the CS gel group and 1,080 are in the placebo gel group), a two-sided log-rank test conducted at the 0.05 significance level has at least 80% power to reject the null hypothesis of no difference in HIV infection rates if the true hazard ratio is 0.5 (i.e., CS gel reduces the HIV infection rate by 50%), the rate of infection in the control group is 5/100 woman-years, and loss to follow-up is at most 20% in both groups. The overall study size has been increased by a factor of 1.008 to allow for the one planned interim analysis using O’Brien-Fleming boundaries (see below). These calculations were conducted using the PASS© 2000 software, which assumes exponential models for the times to HIV detection and losses to follow-up. Approximately 66 total HIV infections are required to achieve the desired power.

## 9.2 Analysis Plan Summary

A detailed analysis plan that covers both the final analysis and the planned interim analysis will be developed and approved prior to the initiation of the study. The following is a summary of the planned analyses. Any deviations to be made from this summary plan will be documented in the detailed analysis plan.

All primary and most secondary analyses will be conducted on an intent-to-treat basis. However, in order to assess the effectiveness and safety of CS among those participants who complied with key aspects of the study protocol and to evaluate the potential impact of treatment on loss to follow-up, various additional analysis populations will be defined in the detailed analysis plan prior to study initiation. Any key decisions regarding the timing of outcomes, inclusion/exclusion in various analysis populations, the appropriateness of test statistics or model assumptions, or any other statistical issues will be made in a blinded review of the data (i.e., blinded to the true randomization groups). Unblinding with respect to the true randomization groups will only be done for the final interpretation of the results, unless otherwise required by the data monitoring committee (DMC) (see section 10.2).

### 9.2.1 Analysis of effectiveness outcomes

The effect of CS on the time to HIV detection will be determined using two one-sided log-rank tests stratified by site. The type one error rate for the one-sided test with alternative hypothesis that CS protects against HIV acquisition will be carried out at the 0.025 level and adjusted to account for the interim analysis described below.[[1]](#footnote-2) Secondarily, proportional hazards regression models will be used to estimate the hazard rate ratio, along with a 95% confidence interval, comparing the CS gel group to the placebo gel group for the HIV outcome, controlling for site and any important baseline prognostic variables. A list of the baseline prognostic variables to be included in the models will be provided in the detailed analysis plan prior to study initiation. The homogeneity of the treatment effect across sites will be investigated by introducing a treatment-by-site interaction term to the model. Crude incidence rates, rate ratios, and 95% confidence intervals will be calculated and will be based on the ratio of the number of new infections to the total length of treatment exposure in the interval. Time to HIV detection, in days, will be computed as the difference between the date of HIV detection, estimated using the midpoint between the dates of the first positive HIV test and the preceding negative HIV test, and the enrollment date, plus one.

For the secondary outcome (time to first gonorrheal or Chlamydial infection), time in analysis will start at the date of the first negative test for GC or CT, which may not correspond to the enrollment date since negative tests for GC and CT are not part of the eligibility criteria. The date of the first infection after the start of time in analysis will be estimated using the midpoint of the study interval in which the first infection was detected. Time to first GC or CT infection will be computed as the difference between the estimated date of the first infection and the start date, plus one. The hazard rate ratio comparing the CS gel group to the placebo gel group for the combined GC or CT outcome will be estimated, along with a 95% confidence interval, using a proportional hazards model, controlling for site and any important baseline prognostic variables. A list of the baseline prognostic variables to be included in the models will be provided in the detailed analysis plan prior to study initiation. No hypothesis tests are planned for the secondary outcome.

In addition to the primary and secondary study objectives, Investigators have a tertiary interest in comparing pregnancy rates between the CS gel and placebo gel groups. Crude pregnancy rates and corresponding 95% confidence intervals will be calculated by randomization group, and stratified by contraceptive method used at baseline; no hypothesis tests regarding the pregnancy outcome are planned. Because this is not a study of the contraceptive effectiveness of CS and because participants will not be discontinued after they develop a desire to become pregnant during the study (which could potentially influence their use of study product), any results regarding this tertiary outcome will be interpreted with extreme care.

### 9.2.2 Analysis of safety outcomes

On a quarterly basis throughout the trial, AEs will be coded using the MedDRA system in a collaborative effort by the study clinician and data management staff. The AEs will be summarized by body system, pooled over treatment arms (i.e., completely blinded). Prevalence and incidence rates, pooled over treatment arms, will be calculated for certain important or interesting AEs or groups of AEs (to be determined in collaboration with project leader and study clinician). These summaries will be used for monitoring purposes only.

At the end of the trial, AEs occurring during the study will be summarized in frequency tables (including both the number of each type of AE and the number of distinct participants with each type of AE), by body system and by randomization group.

## 9.3 Interim Analysis Plan Summary

Two interim analyses for the use of the DMC are planned to evaluate the safety and effectiveness of CS relative to the placebo in terms of preventing HIV infection. The first interim analysis will take place after approximately one quarter of anticipated HIV infections (i.e., 16 infections) have occurred and will focus exclusively on safety (with HIV as the primary safety endpoint), and the second interim analysis will take place after approximately one half of anticipated HIV infections (i.e., 33 infections) have occurred and will evaluate both the safety and effectiveness of CS. Interim analyses will be conducted on a blinded basis as follows. Project biostatisticians will prepare and verify the interim analysis programs using randomly generated dummy treatment assignments that have no relationship to the true treatment assignments. An independent biostatistician not otherwise involved with the study will subsequently incorporate the blinded treatment codes (i.e. “Drug A” or “Drug B”) into the analysis programs for reporting of interim results. The unblinded treatment codes will be provided separately to the DMC by an FHI statistician (the Randomization Manager) who is not otherwise involved in the study. The interim analyses will include descriptive statistics (e.g., frequency distributions for categorical variables and means, standard deviations, medians, and ranges for continuous variables) of baseline variables, compliance with intervention, discontinuation rates, safety data (i.e., adverse events), and STI and HIV incidence. It is recognized that scheduling restrictions of the study monitors and the DMC may preclude performing the interim analyses at precisely the study quarter- and half-way points, and methods will be employed that accommodate these restrictions.

### 9.3.1 Study size assessment

Prior to the second interim analysis (i.e., after approximately 33 events) of the HIV outcome and prior to examining event rates by blinded randomization group, the lead statistician will make recommendations (based on data pooled across randomization groups) regarding adjustment of study size in accordance with the requirement of observing approximately 66 total HIV infections by the conclusion of the study. If either the pooled HIV infection rate or the follow-up rate is substantially lower than anticipated when the study was designed (0.0375 and 0.80, respectively), then consideration will be given to increasing the total study size. Under no circumstances will the statistician recommend decreasing the overall study size. The observed treatment effect at the time of the interim analysis will not be considered as part of this evaluation. Hence no type I error rate adjustment is required to account for this study size assessment.

### 9.3.2 Interim analysis of HIV outcome

Considerations for early termination of the study will be made primarily based on comparisons of the HIV event rates between treatment groups. At the first interim analysis after approximately 16 events, we will ask that the DMC only consider stopping the study for evidence of a potentially harmful CS effect (i.e., evidence that CS enhances HIV transmission), as determined by a one-sided log-rank test at the 0.10 significance level. At the second interim analysis after approximately 33 events, we will ask that the DMC allow consideration for stopping the study either due to evidence of a potentially harmful CS effect, as determined by a one-sided log-rank test at the 0.10 significance level, or due to evidence that CS effectively prevents HIV transmission; the Lan-Demets^16^ spending function with O’Brien-Fleming type boundaries^17^ will be employed to preserve the overall one-sided type I error rate for effectiveness at the 0.025 level, regardless of the timing of the interim analysis (the effective significance level for the interim analysis will be approximately 0.0026 if the interim analysis takes place at approximately the half-way point). Note that the two one-sided tests at the second interim analysis can also be thought of as a single two-sided test with asymmetric tail probabilities. Furthermore, the liberal significance level used to test for potential harm serves a dual purpose in that it also provides the DMC with a conservative guideline to allow consideration for terminating the study due to futility.

# 10.0 MONITORING PLAN SUMMARIES

## 10.1 Clinical Monitoring Plan

Site visits by the FHI clinical monitors will be made in accordance with FHI policy and SOPs. The purpose of the monitoring is to assure the quality and accuracy of data collected on the DCF and entered in the database, and to determine that all regulatory requirements surrounding clinical trials are met.

The Investigator will allow the clinical monitors, the regulatory agencies, and FHI designated persons to inspect study documents (e.g., consent forms, drug distribution forms, DCF) and pertinent clinic records for confirmation of the study data.

Various authorized individuals may visit the study site to audit the progress of this study (e.g., FHI personnel, product manufacturer personnel, regulatory personnel). A site visit log will be maintained at the study site in which all site visits made by authorized individuals are recorded. All clinical records and the DCFs for the participants enrolled in this study will be made available for review by these authorized individuals.

Before the study begins, the clinical monitors will conduct an evaluation visit at each site to determine the suitability of the site for the research study. In addition, there will be a study initiation visit (study start-up), quarterly monitoring visits (to track study progress) and a close-out visit. The overall responsibility of the monitors is to ensure that the study is being conducted according to the protocol, FHI SOPs, ICH/GCP and applicable regulatory requirements.

A detailed monitoring plan will be developed for this study and will be used by all clinical monitors. This plan will specify the responsibilities and qualifications of the identified clinical monitors, back-up provisions, in-house monitoring procedures, and site monitoring visit procedures. All monitoring visits will be documented.

## 10.2 Data Monitoring Committee

FHI will assemble an independent DMC made up of at least three members; we plan to include at least one clinician and one statistician. The DMC will meet once before the study begins and twice during the study. The details for the operation and responsibilities of the DMC will be provided in the DMC Operational Plan.

# 11.0 DATA MANAGEMENT PLAN SUMMARY

A detailed data management plan will be written prior to study initiation. It will be modified if significant changes are required, in order to document how data were handled in the study. The following is a brief summary of the plan.

Study sites will capture clinical data on 2-ply paper data collection forms (DCFs). The Investigator is responsible for the accuracy of data entered on the DCF. The original will be kept in the participant’s original file folder at the site and the other copy will be used as a working copy for data entry and filed in the participant’s working file folder when entry is complete.

The sites will connect to an FHI secure server via the Internet using Citrix software and log directly into FHI's 21 CFR Part 11-compliant data management system. Citrix software allows a user to connect to a host computer and perform processing on the host computer, thus eliminating local software configuration concerns. Once the site data entry staff have logged onto FHI's secure network, they will use the ENTER module of the Clintrial™ data base management system (vendor software provided by Phase Forward, Inc.) The site data entry staff will double enter data (i.e., entry is performed by two independent system users) directly into the Clintrial™ data base. Subsequent data management processing (e.g., query generation, medical coding, data set creation) will take place in the Part 11-compliant environment at FHI.

If data entered on the DCFs are taken from an external source (e.g., laboratory reports, patient records, and participant diary cards), the source documents should be maintained in the participant’s medical chart or study file at the site, and should be available for review.

Data queries will be generated at FHI and sent to the Investigator monthly. The Investigator should answer queries within one week of receipt. The data relevant to the query will be updated in the FHI database based on the Investigator’s written response. Investigators must keep copies of all queries stapled to the appropriate DCFs in the participant’s file.

# 12.0 PROTOCOL VIOLATIONS

Emergency departures from protocol that eliminate an apparent immediate hazard to participants and are deemed crucial for the safety and well being of that participant may be instituted for that participant only by the Investigator. The Investigator will notify the FHI Project Leader and local IRB (if appropriate) in writing as soon as possible and document on the Protocol Violation Form reasons for the violation and ensuing events. Protocol violations may also be identified by the Investigator during the course of the study, the clinical monitor during the periodic and closeout monitoring visits as well as during in-house monitoring. The reporting procedures will be specifically detailed in the monitoring plan. Examples of Protocol Violations:

- Omission or inadequate administration of informed consent
- Inclusion/exclusion errors, including legal age limit
- Treatment errors: no treatment or incorrect treatment (including dose or regimen, expired product)
- Missing or incorrectly timed study procedures and assessments
- Forcing a participant to enter or remain in the study
- Participants who should have been discontinued from the study due to protocol criteria, but were not

# 13.0 STUDY DOCUMENTS

## 13.1 Study Initiation

The following documents will be in place and monitored by a Clinical Monitor at the study site before any potential participants are contacted.

- Investigator’s Brochure
- FDA Form 1572
- Signed protocol and amendments
- Sample DCFs
- Financial disclosure forms for Investigator and Sub-Investigator
- Information given to participants:


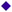
 Consent forms


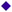
 Other written information


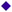
 Advertisements

- Financial records
- Signed agreements between involved parties
- IRB approval
- IRB composition
- Regulatory authority approval
- CV for Investigator, Sub-Investigator, and Site Coordinator
- Laboratory test normal values
- Lab accreditation and lab supervisor’s CV
- Instructions for handling of investigational product and trial related materials
- Shipping records
- Decoding procedures for masked trials
- Site Visit Log

## 13.2 Study Conduct

During the study the following documents will be in place and periodically monitored by a Clinical Monitor at the study site. Revision of documents will be made if relevant.

- Revisions to the Investigator’s Brochure
- Revisions to the FDA form 1572
- Revision to the Financial Disclosure forms
- Revision to the protocol and amendments
- Revisions to the DCFs
- Revisions of information given to participants


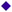
 Consent forms


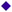
 Other written information


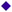
 Advertisements

- IRB approvals
- IRB composition changes
- Regulatory authority approvals
- CV for Investigator, Sub-Investigator, and Site Coordinator
- Updates of laboratory test normal values
- Documentation of investigational product and trial related materials shipment
- Relevant communications other than site visits
- Signed consent forms
- Source documents
- Copies of completed DCFs
- Copies of documentation of DCF corrections
- Notification of Investigator to FHI of SAEs
- Notification of Investigator to local IRB and Regulatory Authorities
- Notification by FHI or Sponsor to Investigator of safety information
- Interim or annual reports to IRB, Regulatory Authorities, and FHI
- Participant screening log
- Participant ID code list
- Participant enrollment log
- Investigational products accountability documents
- Staff signature list
- Site Visit Log
- Progress Reports to FHI

## 13.3 Study Completion

After completion of the study, all of the documents in 13.1 and 13.2 should be in the file together with the following.

- Investigational products accountability documents
- Documentation of products destruction (if destroyed at site)
- Complete participant ID code list
- Final report by Investigator to IRB, Regulatory Authorities, and FHI

## 13.4 Site Record Retention and Access to Documents at the Site

The signed original informed consent documents for each participant, originals and copies of DCFs, and originals of all study documentation (e.g. drug inventory forms, participant clinic records, original laboratory reports, diaries, etc.) will be retained by the Investigator for a minimum of two years after USFDA approval or withdrawal of a New Drug Application (NDA). If an application is not approved, data and document files must be maintained until two years after the US FDA has been notified that shipment and delivery of the product for investigational use is discontinued. **No records may be destroyed without written permission from FHI.**

The Investigator may be subject to a field audit by FHI, CONRAD or FDA inspectors to validate the participation of study participants and to verify the data reported on the DCFs. This audit could occur while the study is in progress, several years after the study is completed, or when the data are under review by the FDA as part of the pre-market approval process. All of the participants’ records and other study documentation must be filed and accessible on short notice (3-5 days) during the study and subsequent retention period.

# 14.0 QUALITY ASSURANCE

All study tests will be conducted locally, by an in-country laboratory (FHI plans to select one laboratory for each site). In order to ensure quality of lab procedures (namely, SDA testing for CT and GC), FHI will put in place a QA plan before initiation of the study. A detailed QA plan will be finalized when the sites and the reference laboratory have been finalized. The reference laboratory will be chosen based on their knowledge, experience and facilities which comply with Good Laboratory Practices (GLP). It may include the following actions:

1. All in-country laboratories participating in the study will be provided with standard laboratory equipment and trained in its use.
2. The study reference laboratory will send a panel of specimens to the in-country lab on a quarterly basis.
3. The in-country laboratory will send a sample of positives and negatives to the study reference laboratory on a quarterly basis for the recheck.
4. The laboratories will receive additional GLP training (if required).

A Quality Assurance Associate from FHI will conduct a site visit and evaluation after at least 5 months of study conduct and then again about 6 months before the projected end of the study. The QA Associate will evaluate study systems for quality control, monitoring of the study, documentation of the study, protocol, GCP and regulatory compliance and overall control of the study.

# 15.0 ETHICS AND RESEARCH INTEGRITY

## 15.1 Institutional Review Board Review and Approval

Before initiating the study, the study must be approved in writing by the Institutional Review Board at the site and FHI’s Protection of Human Subjects Committee in accordance with USFDA regulations (21 CFR Part 56). The study will be conducted in accordance with all conditions of approval by the IRBs. The sites will obtain written reapproval of the research by the IRBs at least annually and forward a copy of the review to FHI.

## 15.2 Informed Consent

No participant may be admitted into this study until the Investigator has obtained her legally effective informed consent. An Investigator shall seek such consent only under circumstances that provide the prospective participant with sufficient opportunity to consider whether or not to participate in the study. Informed consent must be obtained without coercion, undue influence, or misrepresentation of the potential benefits or risks that might be associated with participation in the study.

Informed consent encompasses all oral or written information given to the participant about the study and the study materials. This includes the consent form signed by the participant, recruitment advertising, and any other information provided to the participant. All such information that is given to the participant will be in a language that is understandable to her. The information will not include any language in which the participant is made to waive any of her rights or which releases or appears to release the Investigator, the Investigator’s institution, or FHI from liability for negligence.

Informed consent will be documented by the use of a written consent form that is signed by the participant (or the participant’s mark if she cannot sign). A copy of the signed consent form will be given to each participant. The original signed consent form for each participant will be kept in the participant’s study files separate from the DCFs. The consent form must include each of the basic and additional elements of informed consent described in 21 CFR Part 50.25 and must describe each of the risks or discomforts to the participant that have been identified by FHI as reasonably foreseeable.

## 15.3 Participant Confidentiality

The confidentiality of all participants enrolled into this study will be protected to the fullest extent possible. FHI staff, CONRAD staff, FDA personnel, or other individuals authorized in writing by FHI may audit participant’s clinic records. However, study participants should not be identified by name on any DCF or on any other documentation sent to FHI and will not be reported by name in any report or publication resulting from data collected in this study.

## 15.4 Research Integrity

Concern in the United States about the quality of biomedical and behavioral research has led to the establishment of regulations and guidelines for handling the allegations of scientific misconduct.  As a recipient of U.S. government funding, FHI is required to develop policies and procedures related to scientific misconduct that conform to these regulations.  The regulations define "misconduct" in science as a "fabrication, falsification, plagiarism, or other practices that seriously deviate from those that are commonly accepted within the scientific community for proposing, conducting, or reporting research.  It does not include honest errors or honest differences in interpretations or judgments of data" (42 CFR 50.102.)

FHI has specific obligations under these regulations with respect to the handling of information related to scientific misconduct that may come to its attention.  In addition, the regulations require that FHI establish procedures related to initiating an inquiry, pursuing an investigation, and informing a specified circle of authorities should the situation warrant it.  FHI will pursue all allegations of misconduct in research or questionable academic conduct that may raise legitimate suspicions of misconduct, and will conduct inquiries and investigations to resolve questions regarding the integrity of research.  In conducting inquiries during an investigation, FHI will focus on the substance of the issue and will be vigilant not to permit personal conflicts between colleagues to obscure the facts.

# 16.0 PUBLICATION POLICY

All information concerning the drug supplied by FHI to the Investigator and not previously published is considered confidential.

The initial publication from this study will be the aggregate multicenter results focusing on the primary and secondary endpoints of CS safety, HIV, GC, and CT prevention effectiveness. Subsequent secondary analyses and manuscripts, including any single center data, will be agreed upon by members of the protocol team and reviewed by FHI. All suggestions for the manuscript will be returned to the first author by FHI within 15 working days of receiving it.

For publication of the main findings of this study, the Investigator and Site Coordinator from each site, and the Project Leader and Lead Statistician from FHI will be considered as authors (a total of 6 persons). Other key contributors to the research will be acknowledged as part of the CS Phase 3 Research Team.

# 17.0 ADDITIONAL INVESTIGATOR RESPONSIBILITIES

In addition to the previously described responsibilities, the Investigator is responsible for signing and dating the cover page of this study protocol. The signed and dated original must be submitted to FHI before protocol implementation, and the Investigator at the site must maintain a copy with the study files.

All protocol amendments must be signed and dated by the Investigator. The signed and dated original must be submitted to FHI before implementation of the amendment, and the Investigator at the site must maintain a copy. Amendments must be approved by FHI and the IRB before implementation.

The Investigator will provide FHI with a curriculum vitae (CV) for himself showing the education, training, and experience that qualifies him as an expert in the area of clinical investigation specific to the product under investigation and his affiliation with the site at which the study is being conducted. CVs also must be provided for all individuals listed as Sub-Investigators on the Form FDA-1572 and the Site Coordinator showing the education, training, and experience that qualifies them for their role in the study, and their affiliation with the study site. If and when any personnel listed on the 1572 changes, the Investigator will notify FHI and provide a CV for any new staff on the form.

The Investigator will obtain details of any prior training of the site personnel on human research ethics and send it to the FHI. The Investigator will supply FHI with copies of the current license and/or laboratory certification (such as the Clinical Laboratory Improvement Act [CLIA] certification) of any laboratory used for the study. The Investigator is responsible for obtaining any updates to these documents and sending them to FHI in a timely fashion. This includes documentation of the normal ranges of the laboratory tests used by the laboratory. The Investigator will ensure that appropriate health care or referral is provided for the study participants throughout the study. Copies of all these documents must be maintained at the site.

Throughout the course of the study, the Investigator will prepare and submit to FHI whatever reports are required and detailed in the Subcontract Agreement to be signed prior to initiating the study. These reports summarize the accomplishments of the assignment and usually include the following:

- all changes in the research activity; and
- all unanticipated problems involving risks to participants or others.

The final technical report will be submitted within 45 days after completion of the study unless this period is extended in writing by FHI.

# 18.0 REFERENCES

1. Joint United Nations Programme on HIV/AIDS. Report on the Global HIV/AIDS Epidemic 2002.

2. Wortley PM, Fleming PL. AIDS in women in the United States. Recent trends. JAMA 1997; 278(11):911-6.

3. Haynes BF. HIV vaccines: where we are and where we are going. Lancet 1996; 348(9032):933-7.

4. Centers for Disease Control . Update: barrier protection against HIV infection and other sexually transmitted diseases. MMWR 1993; 42:589-97.

5. Stone AB, Hitchcock PJ. Vaginal microbicides for preventing the sexual transmission of HIV. AIDS 1994; 8:S285-S293.

6. Elias CJ, Heise LL. Challenges for the development of female-controlled vaginal microbicides. AIDS 1994; 8(1):1-9.

7. Elias CJ, Coggins C. Female-controlled methods to prevent sexual transmission of HIV. AIDS 1996; 10:S43-S51.

8. The International Working Group for Vaginal Microbicides. Recommendations for the development of vaginal microbicides. AIDS 1996; 10:UNAIDS1-UNAIDS6.

9. Niruthisard S, Roddy RE, Chutivongse S. The effects of frequent nonoxynol-9 use on the vaginal and cervical mucosa. Sex Transm Dis 1991; 18(3):176-9.

10. Roddy RE, Cordero M, Cordero C, Fortney JA. A dosing study of nonoxynol-9 and genital irritation. Int J STD AIDS 1993; 4(3):165-70.

11. CONRAD. Investigators’ Brochure: Sodium Cellulose Sulfate 6% Vaginal Gel, 2003.

12. Mauck C, Weiner DH, Ballagh S *et al*. Single and multiple exposure tolerance study of cellulose sulfate gel: a Phase I safety and colposcopy study. Contraception 2001; 64(6):383-91.

13. Godts P. ARS Midici 1973; 1055-9 (in French).

14. Martinez Sauser V, Roya AP. El Medico 1984; 91:82 (in Spanish).

15. Mauck C, Frezieres R, Walsh T, Robergeau K, Callahan M. Cellulose sulfate: tolerance and acceptability of penile application. Contraception 2001; 64(6):377-81.

16. Lan KKG, DeMets DL. Discrete sequential boundaries for clinical trials. Biometrika 1983; 70:659-63.

17.O'Brien PC, Fleming TR. A multiple testing procedure for clinical trials. Biometrics 1979; 35:549-56.

# APPENDIX 1: STUDY ACTIVITIES CHART

| **Event** | **Screening** | **Enrollment** | **Follow-Up** | **Final Visit** |
| --- | --- | --- | --- | --- |
| **Informed consent** | ◆ | ◆ |  |  |
| **Eligibility criteria assessment** | ◆ | ◆ | ◆ | ◆ |
| **Assign Participant ID number** | ◆ |  |  |  |
| **Pre-test HIV counseling** | ◆ | ◆ | ◆ | ◆ |
| **HIV test** | ◆ | ◆ | ◆ | ◆ |
| **Syphilis serology** |  | ◆ | (if indicated) | (if indicated) |
| **Urine pregnancy test** | ◆ | ◆ | ◆ | ◆ |
| **Obtain contact information** | ◆ | ◆ | ◆ |  |
| **Pelvic exam** | ◆ | (if indicated) | (if indicated | (if indicated) |
| **Vaginal wet mount** | ◆ | (if indicated) | (if indicated) | (if indicated) |
| **Gonorrhea and Chlamydia SDA** | ◆ | ◆ | ◆ | ◆ |
| **Counseling for STI prevention** | ◆ | ◆ | ◆ | ◆ |
| **Interview** | ◆ | ◆ | ◆ | ◆ |
| **Post-test HIV counseling & test results** | ◆ | ◆ | ◆ | ◆ |
| **AE assessment** |  |  | ◆ | ◆ |
| **Concomitant medications** |  |  | ◆ | ◆ |

# APPENDIX 2: DIRECTIONS FOR USE OF STUDY GEL

**Instructions on Gel Use**

**Please read the following directions carefully before use:**

1) Wash your hands with soap and water before you use the study gel.

2) Use the gel right before you are ready to have sex. If more than one hour has passed since insertion, insert a new applicator of gel.

3) Insert plunger into prefilled applicator by placing small end of plunger into the open hole at the end of the applicator and push until you feel it snap into place. Do not push it past that point.


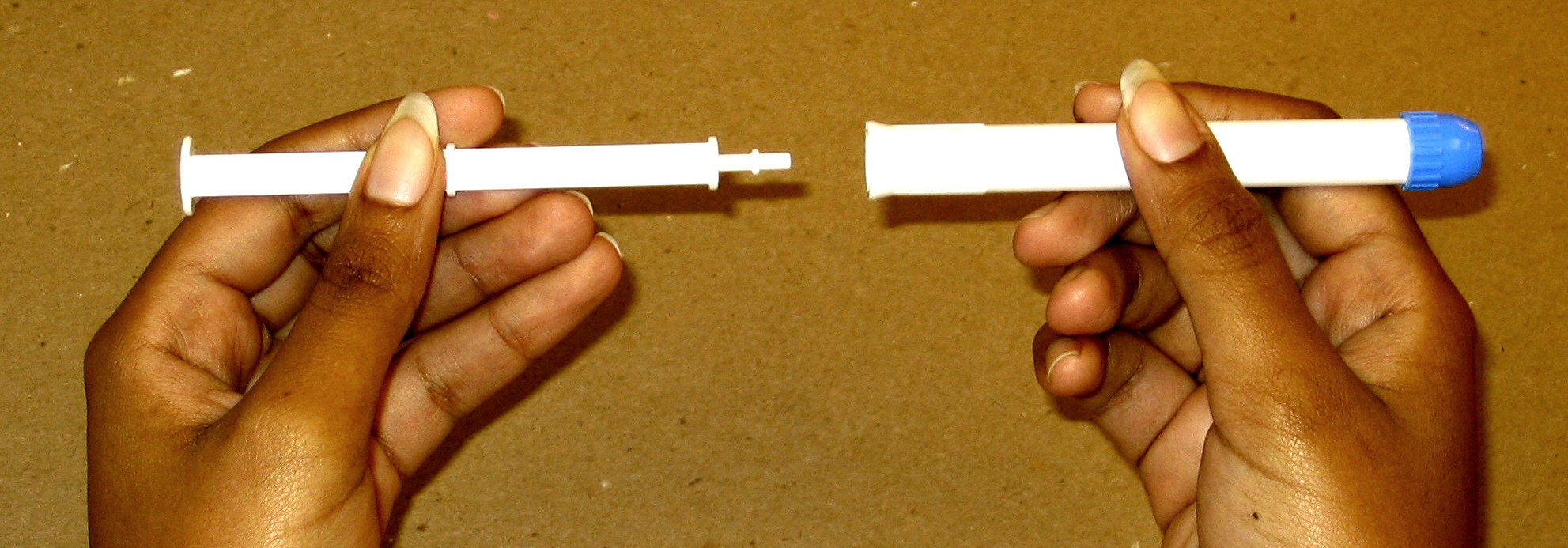


4) Twist to remove the blue cap from the prefilled applicator.

5) Hold the prefilled applicator by the opposite end from the where the gel is. Gently insert the

applicator into the vagina as far as it will go comfortably. Push the plunger in to expel the gel.


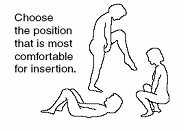

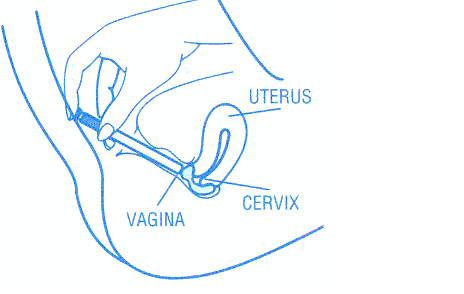


6) Throw away the applicator after each use. Do not flush in toilet.

7) Use a new applicator each time you have vaginal sex, no matter how many time you have sex.

1. The protocol originally described this same testing approach as “two-sided”. However, since the type I error rate will be tightly controlled at 0.025 for detecting a protective effect of the CS gel but will be greater than 0.025 for detecting harmful a effect of the CS gel it is now being described as “two one-sided tests”. [↑](#footnote-ref-2)
